# Genome-wide identification of *GRAS* genes in *Brachypodium distachyon* and functional characterization of *BdSLR1* and *BdSLRL1*

**DOI:** 10.1186/s12864-019-5985-6

**Published:** 2019-08-06

**Authors:** Xin Niu, Shoukun Chen, Jiawei Li, Yue Liu, Wanquan Ji, Haifeng Li

**Affiliations:** State Key Laboratory of Crop Stress Biology for Arid Areas, College of Agronomy, Northwest A&F University, Yangling, 712100 China

**Keywords:** *GRAS*, *Brachypodium distachyon*, Genome-wide analyses, *DELLA*, GA

## Abstract

**Background:**

As one of the most important transcription factor families, GRAS proteins are involved in numerous regulatory processes, especially plant growth and development. However, they have not been systematically analyzed in *Brachypodium distachyon*, a new model grass.

**Results:**

In this study, 48 *BdGRAS* genes were identified. Duplicated genes account for 41.7% of them and contribute to the expansion of this gene family. 33, 39, 35 and 35 *BdGRAS* genes were identified by synteny with their orthologs in rice, sorghum, maize and wheat genome, respectively, indicating close relationships among these species. Based on their phylogenic relationships to *GRAS* genes in rice and maize, *BdGRAS* genes can be divided into ten subfamilies in which members of the same subfamily showed similar protein sequences, conserved motifs and gene structures, suggesting possible conserved functions. Although expression variation is high, some *BdGRAS* genes are tissue-specific, phytohormones- or abiotic stresses-responsive, and they may play key roles in development, signal transduction pathways and stress responses. In addition, *DELLA* genes *BdSLR1* and *BdSLRL1* were functionally characterized to play a role in plant growth via the GA signal pathway, consistent with GO annotations and KEGG pathway analyses.

**Conclusions:**

Systematic analyses of *BdGRAS* genes indicated that members of the same subfamily may play similar roles. This was supported by the conserved functions of *BdSLR1* and *BdSLRL1* in GA pathway. These results laid a foundation for further functional elucidation of *BdGRAS* genes, especially, *BdSLR1* and *BdSLRL1*.

**Electronic supplementary material:**

The online version of this article (10.1186/s12864-019-5985-6) contains supplementary material, which is available to authorized users.

## Background

Transcription factors play key roles in plant growth, development and stress responses. Among them, GRAS proteins are an important family. The acronym, GRAS, originates from the first three functionally characterized *gibberellic acid insensitive* (*GAI*), *repressor of GAI* (*RGA*) and *scarecrow* (*SCR*) genes [1–3]. Subsequently, many *GRAS* genes have been functionally characterized to participate in a number of processes during plant growth and development, including radial organization [3, 4], root development [5, 6], formation and maintenance of meristems [7–13], anther microsporogenesis [14] phytochrome transduction [15–17], gibberellin signaling [1, 2, 18–20], brassinosteroid signaling [21], and responses to stresses [22–24], and other processes.

Most GRAS proteins share a highly conserved GRAS domain in the C-terminal that is composed of five motifs: LHRI (leucine heptads repeat I), VHIID, LHRII (leucine heptads repeat II), PFYRE and SAW [25, 26]. With a leucine-rich repeat, LHRI and LHRII are vital for protein dimerization; VHIID may interact with other proteins [27]. VHIID, PFYRE and SAW are also important for stabilizing the structure of the GRAS domain and maintaining protein function [28–31]. In contrast, the N-terminal is highly variable and can act as bait during molecular recognition events [32]. For example, the N-terminal domain of SCR is required for interactions with LHP1 and other partners and is essential for repression of asymmetric cell divisions [33]. Other GRAS proteins contain an additional conserved DELLA domain and a TVHYNP motif at the N-terminal and are thus referred to as DELLA proteins. Both the DELLA domain and TVHYNP motif are essential for interaction with GID1 in GA-induced ubiquitination and proteasome-mediated degradation of DELLA proteins [34–38].

According to phylogenic analyses, *GRAS* members are initially divided into eight subfamilies in *Arabidopsis thaliana*: SCR, SHR, DELLA, SCL3, PAT1, LlSCL (SCL9), SCL4/7 and HAM [39]. Since then, members of LAS and DLT subfamilies have been identified [21, 26]. To this point, genome-wide analyses of *GRAS* genes have been reported in several species, for example, Arabidopsis [26], tomato (*Solanum lycopersicum*) [40], *Populus* (*Populus euphratica*) [41], and grape (*Vitis vinifera* L.) [42], et al. However, no systemic analyses of GRAS genes have been reported for *Brachypodium distachyon*, one model grass plant with sequenced genome [43].

In this study, we identified and analyzed *BdGRAS* genes at genome-wide. Meanwhile, we characterized the functions of two *DELLA* genes in plant growth. Our study lays a foundation for further study of *GRAS* genes.

## Results

### Identification of *BdGRAS* genes

**Fig. 1:**
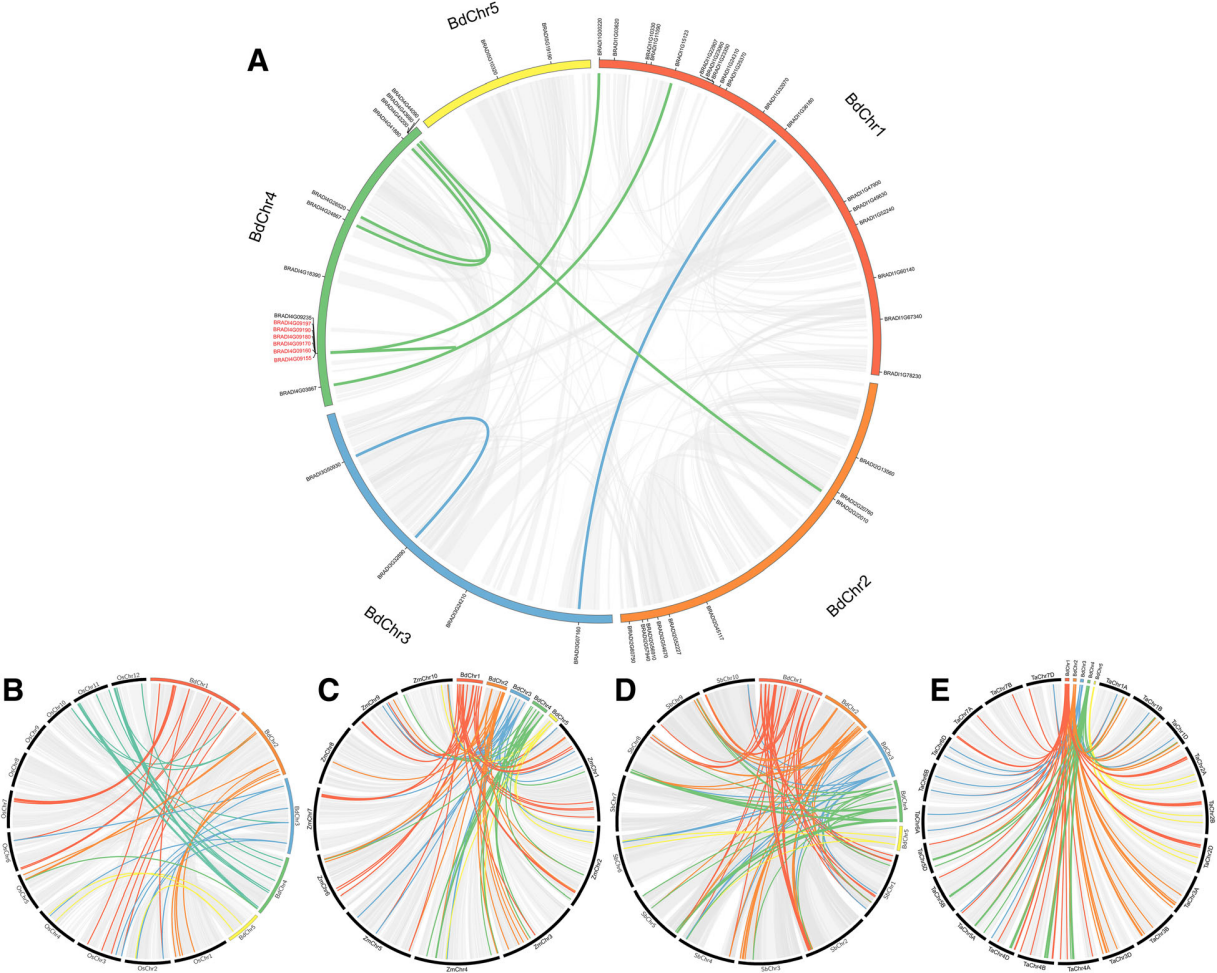
Chromosome location of *BdGRAS* genes and their collinearity with *OsGRAS, ZmGRAS*, *SbGRAS* and *TaGRAS* genes. **a** Chromosome location and duplication of *BdGRAS* genes on *Brachypodium distachyon* chromosomes. (B-E) Duplicated *GRAS* genes between *Brachypodium distachyon*, rice (**b**), maize (**c**), sorghum (**d**), wheat (**e**) genomes. Genes in red were tandem duplicated genes. Chromosomes of *Brachypodium distachyon* were colored in red (BdChr1), orange (BdChr2), blue (BdChr3), green (BdChr4) and yellow (BdChr5). Connecting lines indicate duplicated genes wherein colored lines represent *GRAS* genes while grey lines signify collinear blocks in whole genome

A total of 48 *BdGRAS* genes were identified. This number is more than 33 in Arabidopsis [26], and less than 57 in rice (*Oryza sativa*) [26], and 86 in maize (*Zea mays*) [44]. These genes distribute unevenly on 5 chromosomes (Fig. 1a). Of them, 22 were validated by expressed sequence tags (ESTs) (Additional file 1: Table S1). The length of putative proteins varies from 150 to 805 amino acids with molecular weights (MW) ranging from 16.98 to 88.93 kDa (Additional file 1: Table S1). The grand average of hydropathicity (GRAVY) from 43 BdGRAS proteins was negative while the value of other 5 proteins is close to zero (Additional file 1: Table S1), suggesting that most of BdGRAS proteins were hydrophilic, similar to that in Arabidopsis and *Prunus mume* [45]. The isoelectric point (pI) of BdGRAS proteins varies from 4.77 to 9.95 with an average of 6.27 (Additional file 1: Table S1), implying that most were faintly acidic and different BdGRAS proteins might function in different microenvironments.

### Duplication events and synteny of *BdGRAS* genes

As duplication events have contributed to the expansion of the *GRAS* genes in other plants [41, 42, 44–46], we analyzed the tandem and segmental duplication events of *BdGRAS* genes. 7 (14.6%) *BdGRAS* genes on chromosome 4 were found in a tandem repeat (*BRADI4G09155* through *BRADI4G09197*; Fig. 1a and Additional file 1: Table S2). 14 genes (29.2%, forming 7 segmental duplicated pairs) were identified on four chromosomes (Fig. 1 and Additional file 1: Table S3). Other 28 *BdGRAS* genes showed no corresponding relatives. In total, 41.7% (20/48) of *BdGRAS* genes came from either tandem or segmental duplicated events, indicating an important role for duplication in the expansion of *BdGRAS* genes.

We additionally analyzed the synteny to explore the origin and evolution of *BdGRAS* genes using MCScanX [47]. A total of 32653, 35423, 35346 and 58494 syntenic gene pairs were identified as anchors of collinear blocks between *Brachypodium distachyon* and rice, sorghum, maize and wheat, respectively (Additional file 11: text 1, Additional file 12: text 2, Additional file 13: text 3, and Additional file 14: text 4). This suggests that *Brachypodium distachyon* has significant synteny with these four *Poaceae* and functional studies of genes in *Brachypodium distachyon* may provide information for their homologs. Among them, 33, 39, 35 and 35 *BdGRAS* genes were identified to have orthologs in the corresponding syntenic blocks of rice (Fig. 1b and Additional file 1: Table S4), sorghum (Fig. 1c and Additional file 1: Table S5), maize (Fig. 1d and Additional file 1: Table S6) and wheat (Fig. 1e and Additional file 1: Table S7), respectively. Intriguingly, *TraesCS4A01G176700*/*TraesCS4A01G176600* and *TraesCS4D01G135900*/*TraesCS4D01G136000*, which were homologs of two tandem duplicated gene pairs in *Brachypodium distachyon*, *BRADI4G09155*/*BRADI4G09160* and *BRADI4G09170*/*BRADI4G09197*, respectively, were still tightly linked in the chromosomes of wheat. However, we found no such homologs in other three species, indicating higher conservation of these blocks and a closer relationship between *Brachypodium distachyon* and wheat.

In the grass family, the *Bambusoideae*, *Ehrhartoideae* and *Pooideae* clade split with the *Panicoideae* about 50 Mya [48]. Subsequently, rice in the *Ehrhartoideae*, wheat and *Brachypodium distachyon* in the *Pooideae* split about 46 Mya [48]. Then *Brachypodium distachyon* and wheat diverged about 38 Mya from a common progenitor [49] while maize and sorghum in the *Panicoideae* diverged about 12 Mya [50]. According to the Ks values [51], average divergent time of tandem and segmental duplicated *BdGRAS* genes was about 59.6 Mya and 71.0 Mya, respectively, earlier than the diversification of the grasses (50Mya) [48]. *GRAS* genes in *Brachypodium distachyon* split with those in rice, sorghum, maize and wheat about 58.6, 85.3, 74.4 and 46.0 Mya. These results indicate that large-scale duplications predated the divergence of these species and play a role in the expansion of GRAS gene family.

### Phylogenic trees, conserved motifs and gene structures

**Fig. 2:**
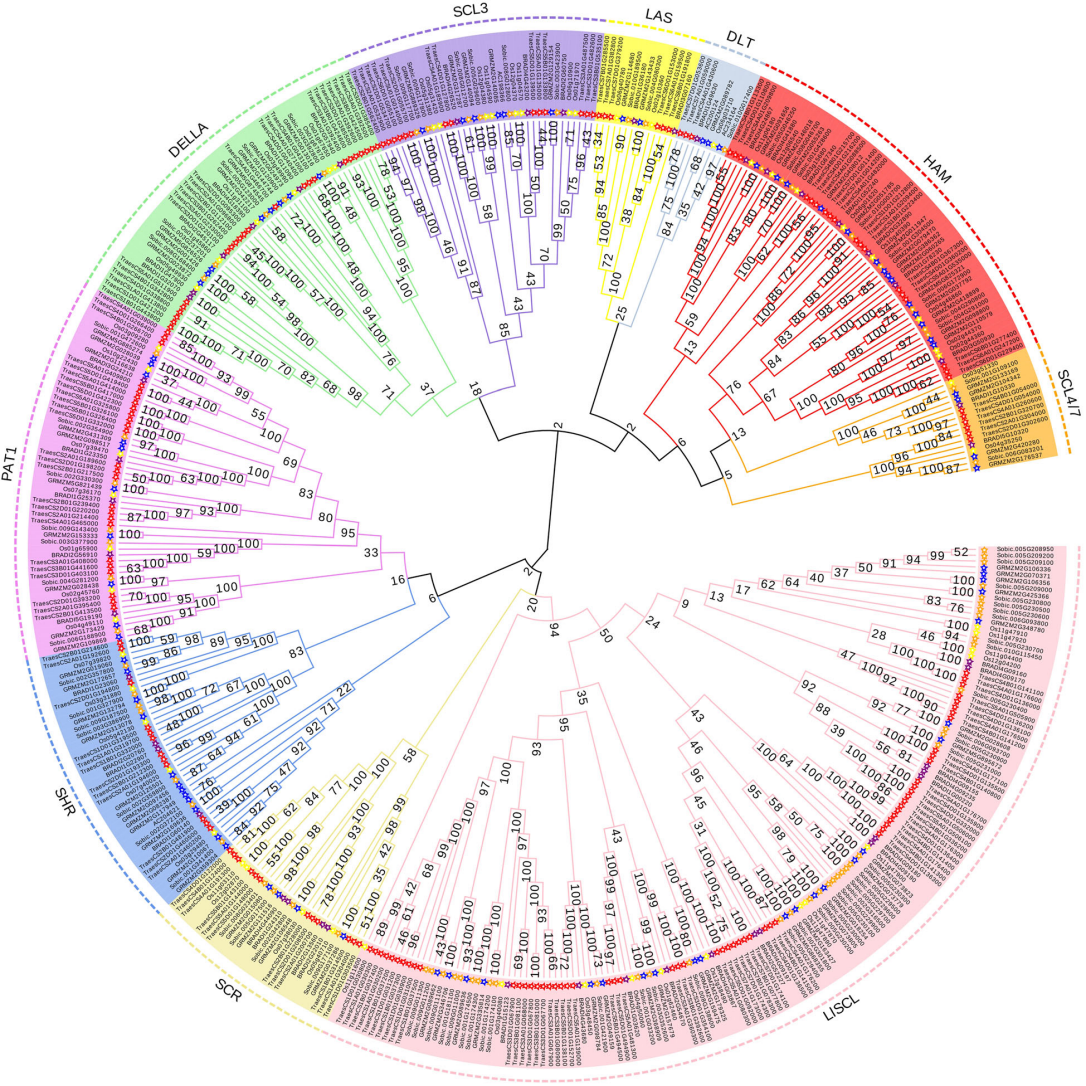
Un-rooted phylogenic tree of GRAS transcription factors in *Brachypodium distachyon*, rice, maize, sorghum and wheat. The GRAS proteins of rice, maize, sorghum, wheat and *Brachypodium distachyon* were marked by yellow, blue, orange, red and purple stars, respectively. Different subfamilies were marked with different background colors

**Fig. 3:**
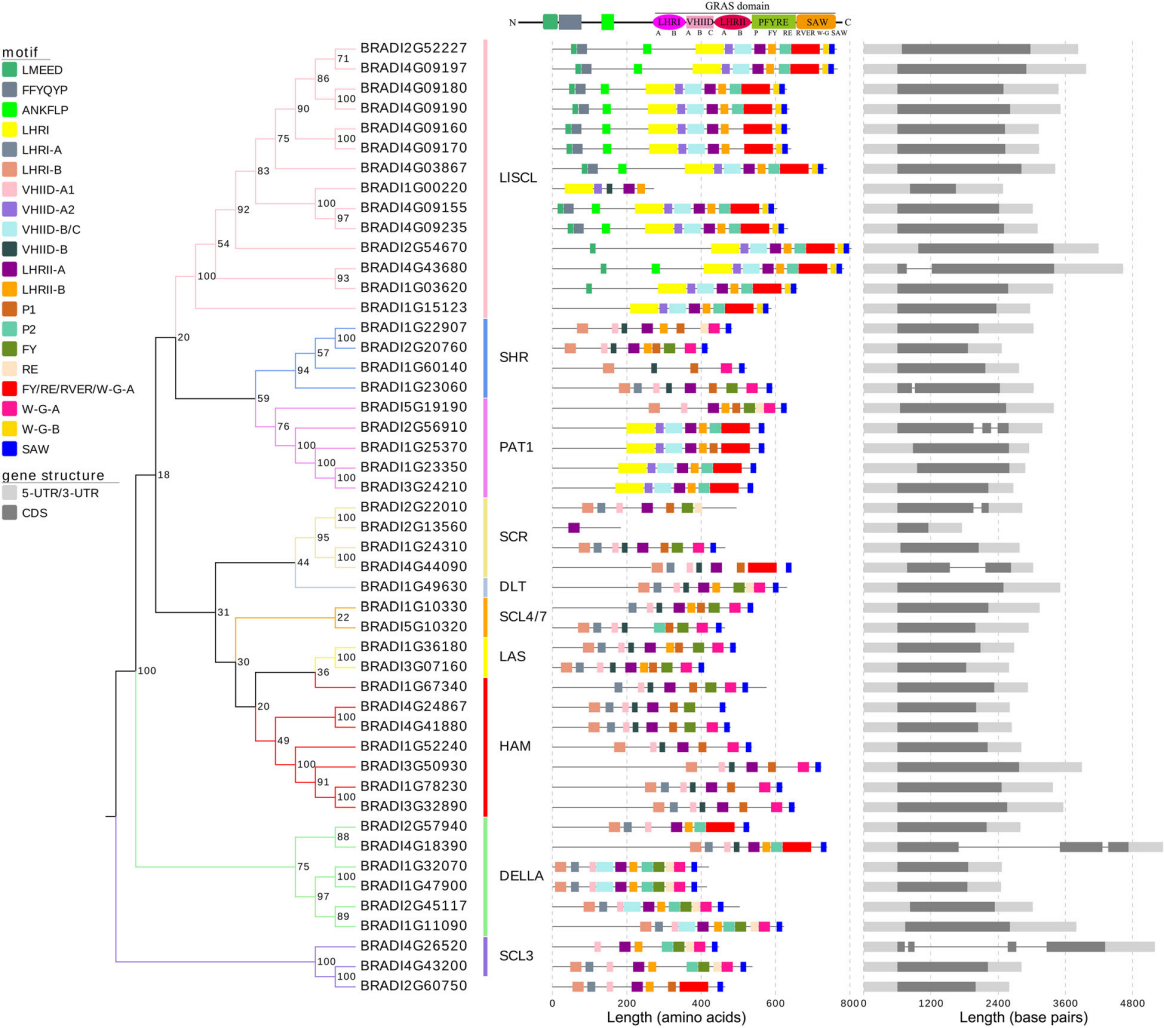
Conserved motifs and gene structures of BdGRAS transcription factors. Different motifs were displayed in different colors; exons and introns were indicated by dark grey boxes and dark grey lines respectively; UTRs were indicated by light grey boxes. The length of motifs, exons, introns and UTRs was drawn in proportion

To study the evolutionary relationships of *BdGRAS* genes, we constructed an un-rooted Neighbor-Joining phylogenic tree (MEGA v6.0 software [52]) based on multiple alignment of 449 GRAS proteins (Additional file 15: text 5) in five grasses including *Brachypodium distachyon* (48), wheat, sorghum (*Sorghum bicolor*) (80) [44], maize (86) [44] and rice (56) [26] (Fig. 2). We also built a second tree based on the multiple alignment of BdGRAS proteins (Fig. 3). Both phylogenic trees showed similar classifications. According to the clade support values and the classification of orthologs in rice and maize [26, 41, 44], *BdGRAS* genes were divided into ten known subfamilies: DELLA (consisting of 6 *BdGRAS* genes), HAM (7), LISCL (14), PAT1 (5), LAS (2), SCR (4), SHR (4), DLT (1), SCL3 (3) and SCL4/7 (2) (Fig. 2 and Fig. 3). Duplicated gene pairs were in the same subfamily. All 7 tandem duplicated genes belonged to subfamily LlSCL, similar to those in grapevine and *Prunus mume* [42, 45]. Segmental duplicated gene pairs were also distributed in the same subfamilies (*BRADI3G32890* and *BRADI3G50930*, *BRADI4G24867* and *BRADI4G41880* in subfamily HAM; *BRADI1G36180* and *BRADI3G07160* in LAS; *BRADI1G00220* and *BRADI4G09155*, *BRADI1G15123* and *BRADI4G03867* in LlSCL; *BRADI4G26520* and *BRADI4G43200* in SCL3; *BRADI2G22010* and *BRADI4G44090* in SCR).

In each subfamily, amino acid sequences of BdGRAS proteins showed high identities. Similar to their homologs in other plants [26, 40, 44, 53], all BdGRAS proteins possess a GRAS domain consisting of LHRI, VHIID, LHRII, PFYRE and SAW at the C-terminal (Additional file 2: Figure S1). In contrast to the conserved C-terminal, the N-terminal of BdGRAS proteins varied substantially while members of the same subfamily possess relatively conserved motifs that might be correlated to different functions [32]. Fourteen subfamily-specific motifs, identified by Sun et al. [32] containing hydrophobic or aromatic residues repeat at the N-terminal, were also found in particular subfamilies of BdGRAS proteins except for LAS proteins with too short N-terminal. These were named after their most conserved amino acids except DELLA, TVHYNP and LR/KXI which were already known (Additional file 2: Figure S1). Motifs NLMAIA and WMESLI exist in BRADI1G10330 in SCL4/7. Motif FLNWI was identified in HAM members except for BRADI1G67340. Motif NVREII was found in BRADI4G44090 and motif DEEG with high proportion of positively and negatively charged residues was detected in BRADI2G22010, BRADI1G24310 and BRADI4G44090 which all belong to subfamily SCR. Motif RAKRT is located in the DLT protein BRADI1G49630. Motif LRSDERG lies in in SCL3 members. Motifs DELLA, TVHYNP and LR/KXI are exclusive to the DELLA member BRADI1G11090. Motif ELEXXLL was detected in PAT1 proteins while motif MDEDF was identified in SHR. Motif YISRMLM and motif FDKVLL were found in most LlSCL members. These motifs at the N-terminal may contain molecular recognition features essential for protein interactions [32]. For example, motif DELLA and TVHYNP, which were exclusive to DELLA subfamily, might directly interact with the GA-receptor GID1a to accept GA signals [38, 54]. Besides, some of these motifs showed rich acidic residues alongside the hydrophobic or aromatic residues, such as motif DELLA, ELEXXLL and YISRMLM, which suggests a connection with transcriptional activation [14, 55]. The distribution of conserved motifs in the N-termini further supported our classification of BdGRAS proteins.

The conserved motifs of full length BdGRAS proteins were identified by MEME. As shown in Figs. 3, 20 conserved motifs were identified (Additional file 3: Figure S2). The majority of the motifs were located in the GRAS conserved domain [26], except for motifs LMEED, FFYQYP and ANKFLP (named after their most conserved amino acids) which were found only in the N-terminal of LlSCL members and appear to be related to transcriptional co-activation functions [32]. Motifs LHRII-A and SAW were found in almost all BdGRAS proteins, indicating high conservation. LHRII-A contained three leucine heptad repeats (LX_6_LX_6_L; L leucine and X any amino acid) that play essential roles in protein interactions [1, 2, 26, 42, 56]. Motif SAW is part of the SAW domain [26] and may be related to stabilizing the structure of the GRAS domain [29].

Other 15 motifs were found only in some BdGRAS proteins. The entire LHRI motif occurred in subfamily LlSCL and PAT1. LHRI-A and LHRI-B were found in other eight subfamilies, indicating a discrepancy in the connected parts between 2 units in these subfamilies. Although both VHIID-A1 and VHIID-A2 were unit A of the VHIID domain, they showed different amino acids and were found in different subfamilies: VHIID-A2 was prominently found in LlSCL and PAT1 while VHIID-A1 was found in other eight subfamilies. VHIID-B was found in nine subfamilies, but not PAT1. Motif VHIID-B/C was found in LlSCL, PAT1, and DELLA, suggesting a closer relationship between these subfamilies. LHRII-B was identified in eight subfamilies except for SCR and HAM. Both P1 and P2 corresponded to the P part of the PFYRE domain; P2 was found in members of subfamily LlSCL, PAT1, DELLA and some members of SCL3 and SCL4/7, while P1 was mainly identified in SHR, PAT1, LAS, SCR, HAM and partial proteins of SCL3 and SCL4/7. Motif FY/RE/RVER/W-G-A contained the FYRE, the RVER and half of the W-G part of the SAW domain, while motifs FY and RE were only part of the PFYRE domain. These three motifs were differently distributed in 10 subfamilies; PAT1, DELLA, SCR and SCL3 contained all three motifs, SHR members contained both FY and RE, proteins in LAS possessed FY/RE/RVER/W-G-A and RE, LlSCL and DLT proteins contained only FY/RE/RVER/W-G-A, and HAM and SCL4/7 members had only FY. As the former and latter part of W-G in the SAW domain, W-G-A was found in nine subfamilies except LlSCL which possessed W-G-B exclusively. In total, GRAS proteins in the same subfamily showed similar motif components and distribution.

Apart from motifs, the gene structures of *BdGRAS* are also quite conserved (Fig. 3). Most *BdGRAS* genes (41/48) are mono-exonic, similar to other reported plant species [40–42, 45]. These intron-less genes could be inherited from ancient prokaryotes [57]. Among the other 7 genes, 4 (2 in subfamily SCR, 1 in subfamily LlSCL and 1 in subfamily SHR), 2 (1 each in subfamily DELLA and SHR), and 1 (in subfamily SCL3) contain two, three and four exons, respectively (Fig. 3). Both tandem duplicated and segmental duplicated *BdGRAS* gene pairs showed similar intron-exon structures except for 2 genes in LlSCL, *BRADI1G15123* (without intron) and *BRADI4G03867* (one intron), which might result from intron gain or loss events [58].

### Expression profiles of *BdGRAS* genes

**Fig. 4:**
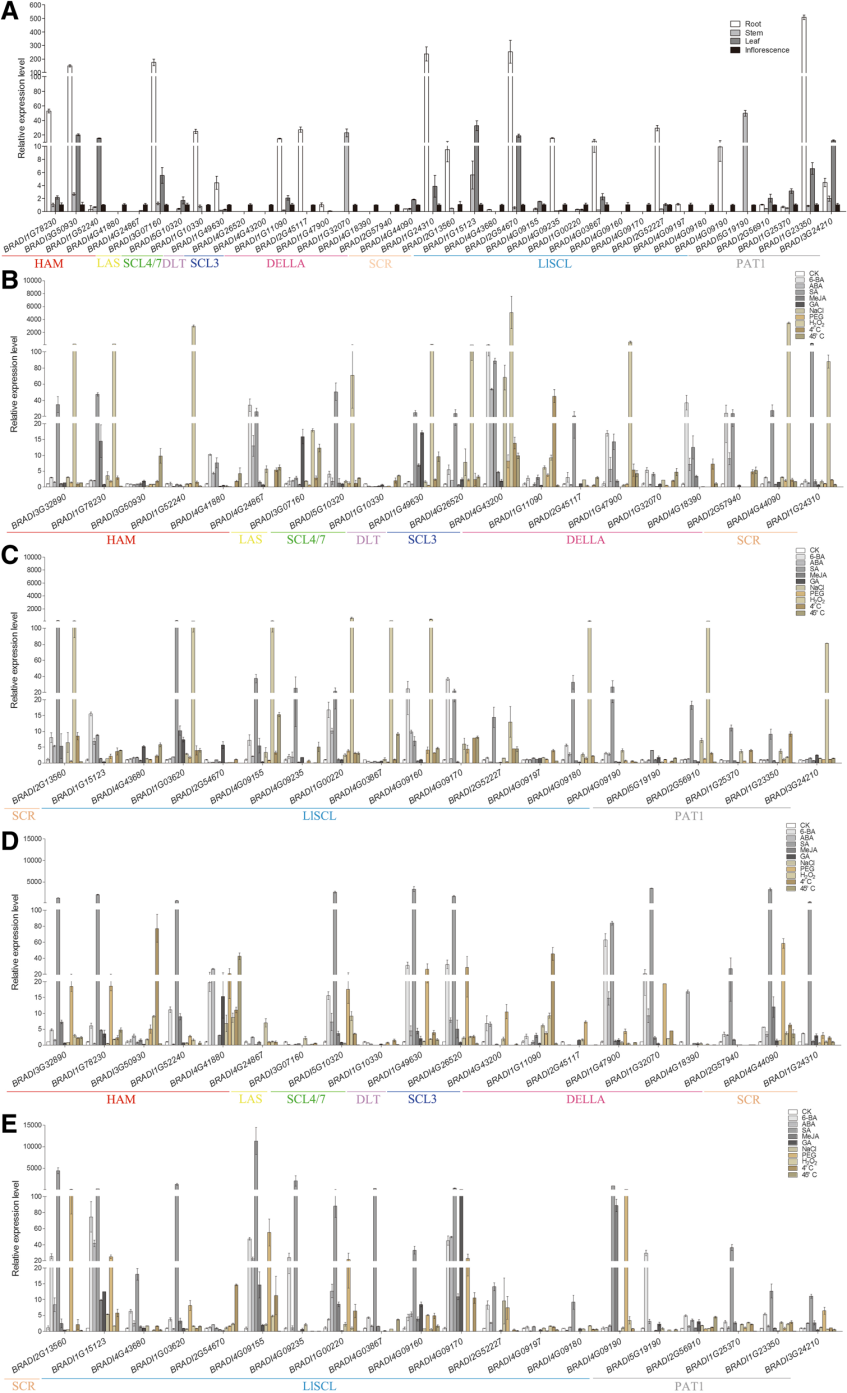
Expression profiles of *BdGRAS* genes in different tissues and under treatments of different abiotic stresses and phytohormones. **a** Expression of *BdGRAS* genes in roots, stems, leaves and inflorescences. (**b-d**) Expression of *BdGRAS* genes in two-week-old seedling leaves (**b** and **c**), roots (**d** and **e**) treated with various phytohormones or under abiotic stresses. Standard errors are indicated by vertical bars

As gene function are related to expression, we analyzed *BdGRAS* genes expression profiles in roots, stems, leaves and inflorescences during the filling stage using qPCR (Fig. 4a). Transcripts of 38 genes were detected (primers are listed in Additional file 1: Table S8). In general, the transcription levels of BdGRAS genes in different tissues varied greatly. Transcripts of 14 genes in seven subfamilies were detected in all four tissues while some genes displayed some tissue-specific expression. For example, *BRADI1G23350* (PAT1) was mainly expressed in roots, *BRADI1G32070* (DELLA) and *BRADI5G19190* (PAT1) were predominantly detected in stems, BRADI1G52240 was highly accumulated in leaves, while *BRADI4G26520* and *BRADI4G43200* (SCL3), *BRADI2G57940* and *BRADI4G18390* (DELLA), *BRADI4G09160*, *BRADI4G09170*, and *BRADI4G09180* (LlSCL), were specifically expressed in inflorescences. Remarkably, the expression of almost all *BdGRAS* genes were detected in inflorescences (except *BRADI1G47900*) which is similar to the expression profiles of *SlGRAS* genes [40]. Tissue specific expression imply that these genes might be involved in these tissues development.

Some *BdGRAS* genes on the same branches showed similar expression profiles. For instance, *BRADI1G23350* and *BRADI3G24210* (PAT1) are mainly expressed in roots and leaves. *BRADI1G25370* and *BRADI2G56910* (PAT1) are expressed in all four tissues with high level in leaves. *BRADI4G26520* and *BRADI4G43200* (SCL3), *BRADI2G57940* and *BRADI4G18390* (DELLA) are specifically expressed in inflorescences. In particular, duplicated gene pairs showed similar expression profiles. For example, tandem duplicated genes (LlSCL) *BRADI4G09160*, *BRADI4G09170*, *BRADI4G09180* are specifically expressed in inflorescences, while *BRADI4G09190* and *BRADI4G09197* are mainly expressed in roots and inflorescences. As segmental duplicated genes, *BRADI4G24867* and *BRADI4G41880* (HAM), *BRADI4G26520* and *BRADI4G43200* (SCL3) accumulated significantly in inflorescences, while *BRADI1G00220* and *BRADI4G09155* (LlSCL) are expressed in leaves, stems and inflorescence. Similar expression patterns may indicate similar functions of these genes in the development of these tissues.

We also investigated gene expression under abiotic stresses and phytohormone treatments. The expression of 40 *BdGRAS* genes was detected. In general, there is no regularity (Fig. 4b-e).

In seedling leaves, 6-BA induced the expression of most *BdGRAS* genes in subfamily DELLA, DLT, SCR, HAM and LlSCL while inhibited the expression of members in subfamily PAT1. In seedling roots, 6-BA induced most *BdGRAS* genes (except *BRADI2G45117* in DELLA, *BRADI1G10330* in SCL4/7, *BRADI3G07160* in LAS, *BRADI4G24867* in HAM, and *BRADI3G24210* in PAT1). Of them, the most up-regulated gene was *BRADI4G43200* in leaves, and the most down-regulated gene was *BRADI2G45117* in roots. In seedling leaves, ABA inhibited the expression of 5 *BdGRAS* genes while up regulated 7 *BdGRAS* genes but have no significant effect on the other 28 genes. In seedling roots, ABA slightly induced 26 *BdGRAS* genes while reduced the expression of 9 genes. Of them, *BRADI4G43200* and *BRADI3G07160* were the most up-regulated and down-regulated genes, respectively. SA up-regulated the expression of 35 leaf and 32 root *BdGRAS* genes, in which *BRADI4G09155* increased a surprising 11287 times in roots. GA suppressed the expression of 23 leaf and 22 root *BdGRAS* genes, wherein *BRADI4G18390* and *BRADI4G09170* declined to zero in both roots and leaves. Some genes expressed differently responding to the same hormone in different tissues. For example, MeJA suppressed the transcription of 21 *BdGRAS* genes in leaves while induced 30 *BdGRAS* genes in roots. GA intensively suppressed the expression of *BRADI3G07160* in roots but induced it in leaves by approximately 16 times. 6-BA promoted *BRADI4G24867* in leaves whereas strongly suppressed it in roots. These results indicated that *BdGRAS* genes might participate in the crosstalk among phytohormones.

The effects of abiotic stresses including salt, drought, oxidation, cold and heat on the expression of *BdGRAS* genes were also detected. NaCl slightly up regulated the expression of 28 leaf and 22 root *BdGRAS* genes. The most up-regulated gene was *BRADI4G43200* in leaves while the most down-regulated gene was *BRADI2G57940* in leaves, *BRADI1G32070* in roots and *BRADI4G09235* in both tissues. PEG promoted the transcription of 21 and 26 *BdGRAS* genes in leaves and roots, respectively. Among these, *BRADI4G09190* in roots increased the most, while *BRADI4G24867* in roots, *BRADI4G03867*, *BRADI4G43680* and *BRADI5G19190* in leaves, and *BRADI2G57940* in both tissues dropped to zero. H_2_O_2_ dramatically increased the expression of 22 *BdGRAS* genes in leaves with the most up-regulated gene expression (3831 times) in *BRADI4G43200*. Twenty genes were slightly induced in roots. Thirty-five leaf and 27 root *BdGRAS* members were induced by cold. In this case, the highest expression was found in *BRADI3G50930* in roots while the lowest in *BRADI2G45117*, *BRADI4G18390*, *BRADI4G43200*, *BRADI4G03867* and *BRADI5G19190* in roots. Heat stress up-regulated the expression of 28 *BdGRAS* genes in leaves while inhibited 26 *BdGRAS* genes in roots. The expression of *BRADI4G41880* in roots was the most elevated while *BRADI3G07160*, *BRADI4G24867* and *BRADI4G09170* in roots declined the greatest. Although they differed greatly, the expression patterns of *BdGRAS* genes identified some tissue-specific genes, phytohormone- and abiotic stress-responsive genes and provided useful information for functional studies.

### *cis*-elements of *BdGRAS* genes

We also analyzed the *cis-*elements of *BdGRAS* genes (Additional file 1: Table S11). Ten *cis-*elements that were related to plant growth and development were identified. Among them, three are light responsive and two are involved in endosperm expression. The remaining are related to meristem expression, circadian control, meristem specific activation, zein metabolism regulation, and cell cycle regulation. Especially, all *BdGRAS* genes contain light responsive *cis-*elements and 43 *BdGRAS* genes contain at least one *cis-*element related to endosperm expression. 19 *BdGRAS* genes contain meristem expression-related cis-element CAT-box. 28 *BdGRAS* genes have the meristem specific activation element CCGTCC-box. 34 *BdGRAS* genes contain circadian control element These results indicate that *BdGRAS* genes participate extensively in plant growth and development.

Ten *cis-*elements were identified to be responsive to different phytohomones including ABA (ABRE was found in 30 *BdGRAS* genes), MeJA (TGACG-motif in 32 *BdGRAS* genes), SA (TCA-element and SARE in 24 *BdGRAS* genes), auxin (AuxRR-core and TGA-element in 17 *BdGRAS* genes), gibberellin (GARE-motif, P-box and TATC-box in 29 *BdGRAS* genes), ethylene (ERE in 8 *BdGRAS* genes). These *cis-*elements may be associated with the expression profile. For example, *BRADI1G32070*, *BRADI1G49630*, and *BRADI4G09190* possessing TCA-element or SARE are strongly induced by SA. ABA positively regulates the expression of *BRADI4G18390* and *BRADI2G13560* that contain ABRE elements. MeJA noticeably inhibits the expression of *BRADI2G45117*, *BRADI1G10330* and *BRADI2G52227* that possess TGAC-motifs. GA inhibits the expression of genes in subfamily DELLA (*BRADI1G11090*, *BRADI4G18390*, and *BRADI2G57940*) and PAT1 (*BRADI1G25370* and *BRADI1G23350*) which have the gibberellin responsive elements TATC-box, P-box or GARE. In total, *cis*-elements correspond with the expression of many *BdGRAS* genes.

### GO annotations and KEGG pathways of BdGRAS proteins and conserved functions of *BdSLR1* and *BdSLRL1* in plant growth

Only one *BdGRAS* gene *BdSHR* has been functionally characterized in *Brachypodium distachyon*. It plays a similar role with its orthologs *AtSHR* and *OsSHR* in the regulation of meristem and root growth [59]. The functions of most *BdGRAS* genes still remain to be studied.

We analyzed the gene ontology of 48 *BdGRAS* genes. Although they were all GO annotated and presumed to be involved in DNA-templated transcription (Additional file 1: Table S9 and S10), no conclusive results were found.

We also performed KEGG pathway analyses of *BdGRAS* genes. Only two genes *BRADI1G11090* (*BdSLR1*) and *BRADI2G45117* (*BdSLRL1*) were identified with the same annotation K14494. Congruent with GO analyses, both genes may be involved in GA mediated signaling transduction pathway.

Based on phylogenic analyses, both *BdSLR1* and *BdSLRL1* were *DELLA* genes whose orthologs in Arabidopsis, rice, maize and wheat play key roles in plant growth via negatively regulating GA signal [1, 2, 20, 28, 34]. However, such functions have not yet been reported in *Brachypodium distachyon*. Here, we characterized the functions of these two *BdDELLA* genes by ectopic expressing them in Arabidopsis.

**Fig. 5:**
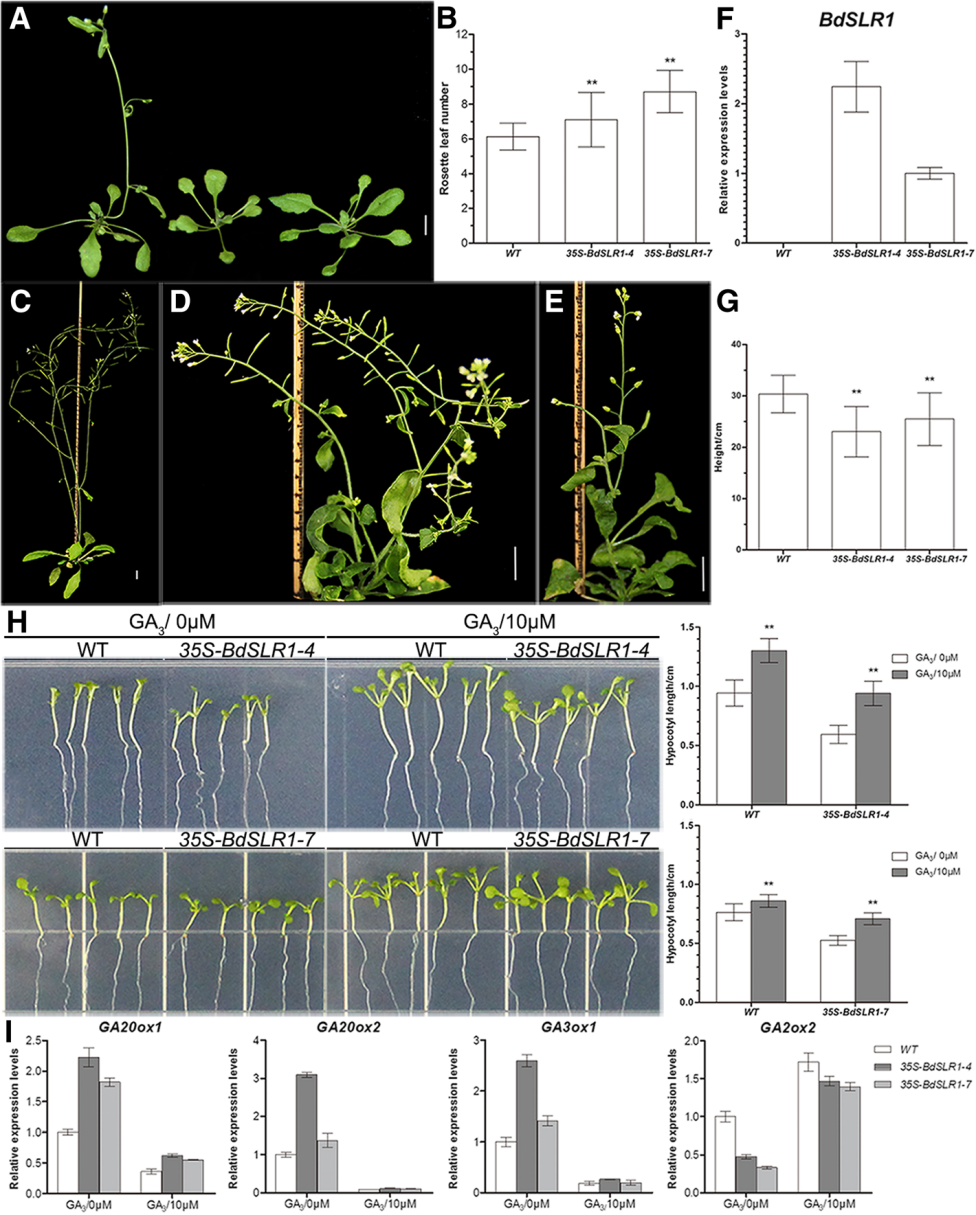
Phenotypes of transgenic Arabidopsis over-expressing *BdSLR1* under normal conditions and GA_3_ treatment. **a** 4-week-old wild type (left) and transgenic Arabidopsis (middle and right). **b** Rosette leaf numbers of wild type and transgenic Arabidopsis at bolting stage. For *35S-BdSLR1–4*, *n* = 33; for *35S-BdSLR1–7*, *n* = 32. **c-e** 6-week wild type (**c**) and two severely dwarf transgenic Arabidopsis (**d** and **e**). **f** Relative expression level of *BdSLR1*. **g** Final height. **h** 7-day-old seedlings and hypocotyl length with 0 μM or 10 μM GA_3_, *n* = 30. **i** Expression levels of GA related genes in wild type and transgenic Arabidopsis. Scale bars = 1 cm. ** indicates that *p*<0.01 by Student’s t test. Standard errors are indicated by vertical bars

Twenty-three transgenic Arabidopsis lines over-expressing *BdSLR1* were obtained. Sixteen lines showed later flowering (Fig. 5a, b) and dwarfism (Fig. 5c-e) compared with the wild type. According to the expression level (Fig. 5f), Lines 4 and 7 were selected for further analyses. After maturation, the height of control and the transgenic plants were measured. The average height of lines *35S-BdSLR1–4* and *35S-BdSLR1–7* was 23.04 ± 4.89 cm (*n* = 33) and 25.47 ± 5.10 cm (*n* = 32) respectively, while the average height of control was 30.34 ± 3.66 cm (*n* = 36) (Fig. 5g). The hypocotyls of transgenic Arabidopsis were clearly shorter than the control (Fig. 5h). These phenotypes are similar to some GA-deficient mutants. When treated with 10 μM GA_3_, the hypocotyl lengths of both transgenic and wild type Arabidopsis increased noticeably (*P* < 0.05), and the rate of transgenic Arabidopsis (58.72%) was higher than that of wild type (38.08%) (Fig. 5h).

We also detected the expression of GA related genes. *GA20-oxidase 1* and *2* catalyze the sequential oxidation of active GAs [60], *GA3-oxidase 1* catalyzes the last step for the synthesis of bioactive GAs [61], and *GA2-oxidase 1* inactivates GA [62]. These four genes are under the feedback regulation of GA; the expression of *GA20ox1*, *GA20ox2* and *GA3ox1* is down-regulated while the expression of *GA2ox*1 is up-regulated by GA_3_ [63]. qRT-PCR results showed that the expression level of *GA20ox1*, *GA20ox2,* and *GA3ox1* was higher than control, while the expression of *GA2ox1* in transgenic Arabidopsis was lower than the control (Fig. 5i). When treated by GA_3_, the expression level of these four genes in transgenic plants recovered to normal levels and was indistinguishable from that of the wild type (Fig. 5i). These results indicated that, as with the GO annotation, *BdSLR1* participates in plant growth via negatively regulating GA signals like its orthologs in other plants [1, 2, 20, 28, 34]..

**Fig. 6:**
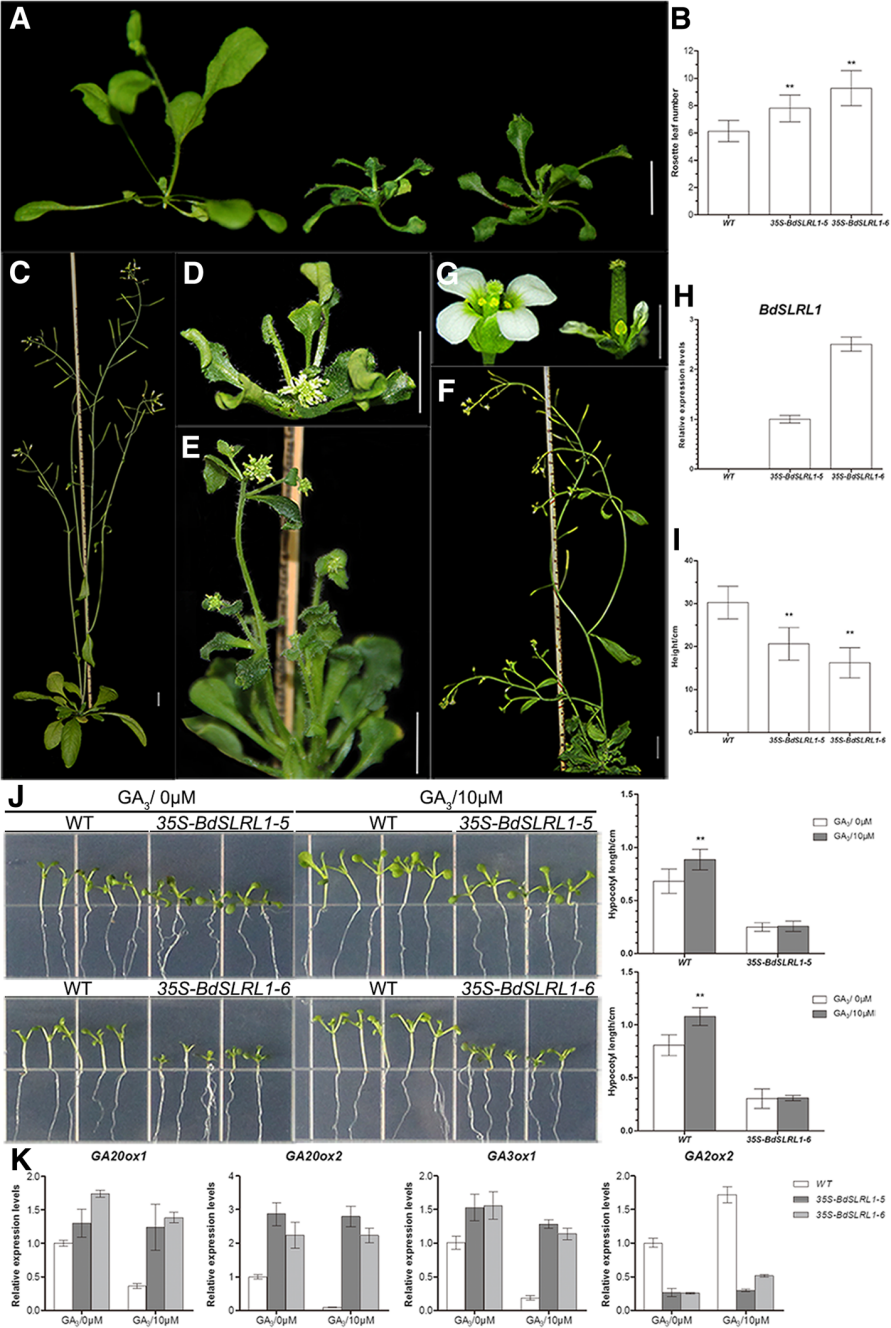
Phenotypes of transgenic Arabidopsis over-expressing *BdSLRL1* under normal conditions and GA_3_ treatment. **a** 4-week-old wild type (left) and transgenic Arabidopsis (middle and right). **b** Rosette leaf numbers of wild type and transgenic Arabidopsis at flowering time. For *35S-BdSLRL1–5*, *n* = 36; for *35S-BdSLRL1–6*, *n* = 31. **c-f** 6-week-old wild type (C) and three typical transgenic Arabidopsis lines (**d-f**). **g** Fower of wild type (left) and short-stamen transgenic Arabidopsis (right). **h** Relative expression level of *BdSLRL1*. **i** Final height. **j** 7-day-old seedlings and hypocotyl length with 0 μM or 10 μM GA_3_, *n* = 30. **k** Expression levels of GA related genes in wild type and transgenic Arabidopsis. Scale bars = 1 cm in A-F; scale bar = 2 mm in G; ** indicates that *p*<0.01 by Student’s t test. Standard errors are indicated by vertical bars

Seventeen transgenic Arabidopsis lines over-expressing *BdSLRL1* were acquired with Fourteen lines displayed late flowering (Fig. 6a, b) and dwarfism (Fig. 6c-f). Among these, four showed a severe dwarf phenotype with a height of less than 3.5 cm (Fig. 6d). Three showed mild dwarfism with a height between 3.5 and 10 cm (Fig. 6e). The height of seven lines was more than 10 cm yet slightly shorter than that of control (Fig. 6f). Additionally, the severe and mild dwarf plants had shorter stamen filaments (Fig. 6g) resulting in sterile flowers. So, the two slightly dwarf lines (Lines 5 and 6) were selected for further analyses due to their expression levels (Fig. 6h). After maturation, the average height of *35S-BdSLRL1–5* and *35S-BdSLRL1–6* transgenic Arabidopsis was 20.61 ± 3.81 cm (*n* = 36) and 16.22 ± 3.48 cm (*n* = 31) respectively, while the average height of control was 30.24 ± 3.80 cm (*n* = 33) (Fig. 6i). The hypocotyls of transgenic Arabidopsis were also significantly shorter (Fig. 6j). The transcription levels of GA-related genes were also similar to those in transgenic Arabidopsis over-expressing *BdSLR1*. Under normal conditions, the expression of *GA20ox1*, *GA20ox2,* and *GA3ox1* was higher, while the expression of *GA2ox1* was lower in transgenic Arabidopsis than that in the wild type. But different from *BdSLR1*, transgenic Arabidopsis that over expressed *BdSLRL1* was insensitive to exogenous GA_3_ (Fig. 6j). Consistent with other findings, exogenous GA_3_ had no obvious effect on the expression of those 4 genes in transgenic plants over-expressing *BdSLRL1* (Fig. 6k). The phenotypes of dwarfism and insensitivity to exogenous GA_3_ in transgenic Arabidopsis over-expressing *BdSLRL1* were also found in transgenic plants over-expressing its orthologous *OsSLRL1* [64] and *OsSLRL2* [65].

These results indicated that both *BdSLR1* and *BdSLRL1* play a conserved role in plant growth via negatively regulating GA signal like their orthologs in Arabidopsis, rice, maize and wheat [1, 2, 20, 28, 34, 64, 65] and verified the GO and KEGG pathway annotations.

### Conserved functional mechanisms of *BdSLR1* and *BdSLRL1*

**Fig. 7:**
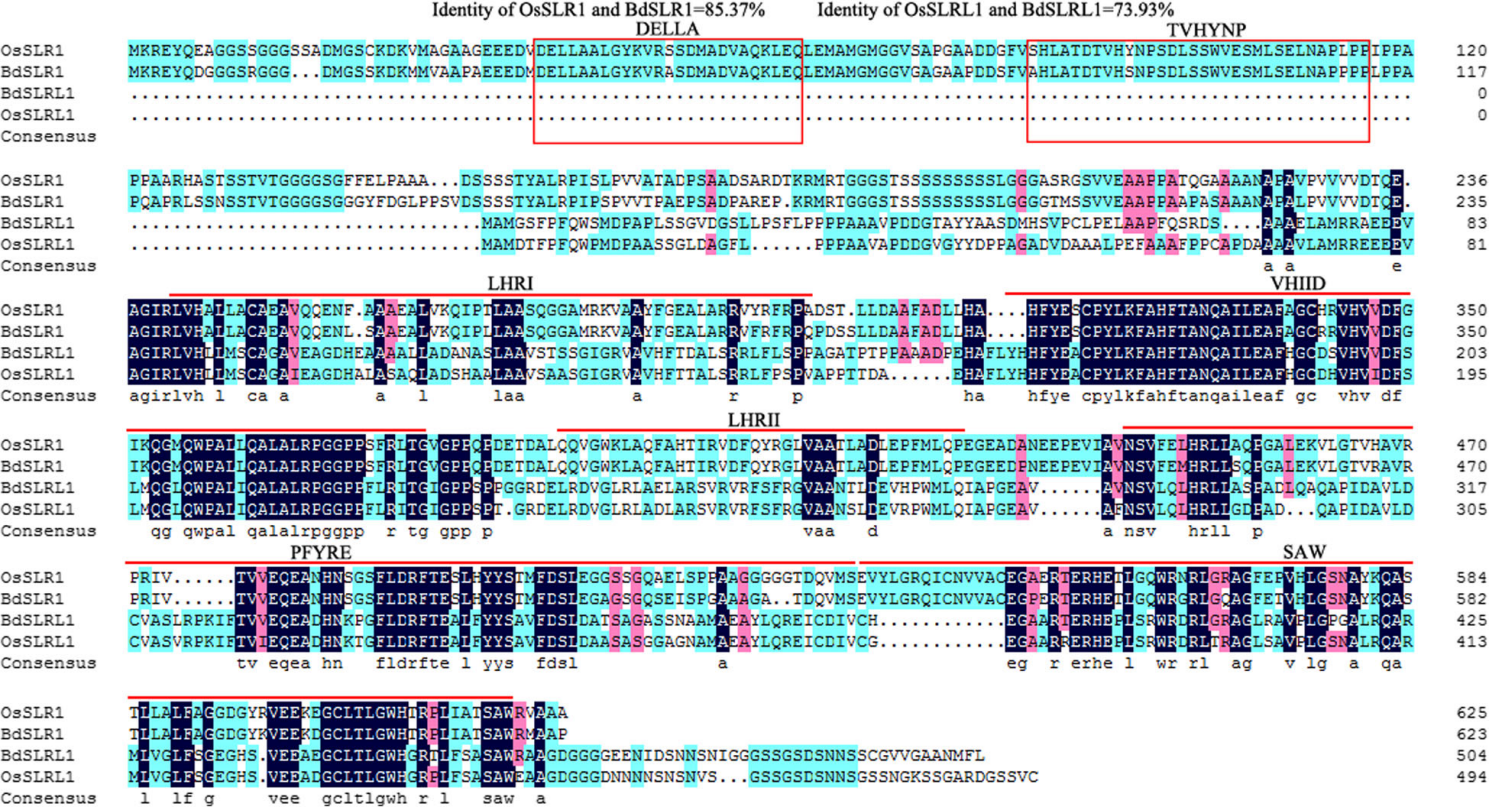
Multiple alignment of BdSLR1, BdSLRL1, OsSLR1 and OsSLRL1 protein sequences. Black, pink and blue background color indicate the homology level of amino acid residues is 100%, above 75, and 50%, respectively. Conserved DELLA domain and TVHYNP motifs were marked with red square

Although *BdSLR1* and *BdSLRL1* regulate plant growth via inhibiting GA mediated signaling pathway, transgenic plants over-expressing two genes showed different sensitivity to exogenous GA_3_, implying some differences between the two genes. As protein is the main manifestation of gene function, we further analyzed both BdSLR1 and BdSLRL1 proteins. Sequence alignment showed that BdSLR1 and BdSLRL1 were highly homologous with OsSLR1 (identity of full length sequence = 85.37%) and OsSLRL1 (identity of full length sequence = 73.93%), respectively (Fig. 7). All four proteins contain a conserved GRAS domain at the C-terminal. In addition, BdSLR1 and OsSLR1 contain an additional DELLA domain and TVHYNP motif at the N-terminal while BdSLRL1 and OsSLRL1 do not. As there may be annotation errors that could lead to a truncated gene sequence, we performed the 5′-RACE of *BdSLRL1* and acquired a single product of 233 bp (Additional file 4: Figure S3 and Additional file 5: Figure S4). A PCR of a full length of BdSLRL1 with 5′-UTR using BdSLRL1FLPF (the first 21 bp of 5′-UTR) and BdSLRL1FLPR (BdSLRL1Y2H reverse primer without a restriction enzyme cutting site) also generated a single band (Additional file 6: Figure S5, Additional file 7: Figure S6, and Additional file 8: Figure S7). Sequence analyses showed that, *BdSLRL1* generates a single transcript of 1604 bp including 89 bp 5′-UTR (Additional file 9: Figure S8), indicating that BdSLRL1 has no DELLA domain or TVHYNP domain.

In rice, SLR1 showed transcriptional activation activity and interaction with GID1 depending on the presence of the DELLA domain and TVHYNP motif [34]. We further investigated whether BdSLR1 and BdSLRL1 have similar activities. Experiment with yeast have shown that BdSLR1 have transcriptional activation activity (Additional file 10: Figure S9). Similar to OsSLR1, after deleting the DELLA domain and TVHYNP motif (1–142 amino acids at the N-terminal), the truncated protein (BdSLR1D) lost the activity, suggesting that the DELLA domain and TVHYNP motif are essential for transcriptional activation of BdSLR1 [34]. Whereas, BdSLRL1 which lacks the DELLA domain and the TVHYNP motif did not show transcriptional activation (Additional file 10: Figure S9).

**Fig. 8:**
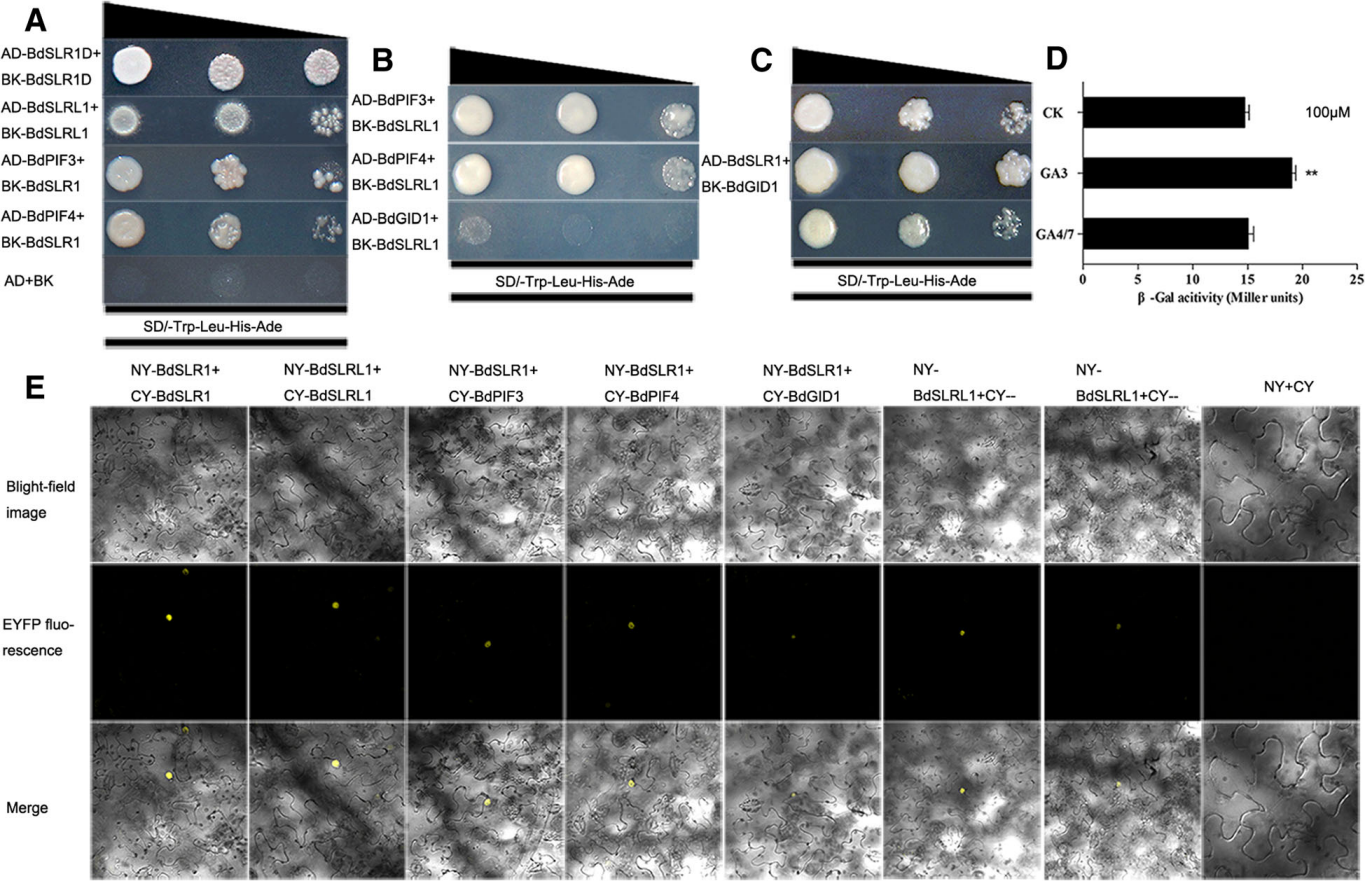
Protein interaction assays of BdSLR1 and BdSLRL1 with BdGID1, BdPIF3 and BdPIF4 and between themselves. **a**, **b** protein interactions in yeast cells (**c**, **d**) enhanced interaction between BdSLR1 and GID1 by GA_3_. Values for ±standard errors were determined by three replicates and ** indicates that *p*<0.01 by Student’s t test. Standard errors are indicated by horizontal bars. **e** Further assay of protein interactions by BiFC in tabacoo leaf cells

RGA (an ortholog of BdSLR1 in Arabidopsis), which can be degraded by interacting with GID1 [66, 67], negatively controls PIFs-mediated hypocotyl elongation through physically interacting with phytochrome-interacting factors (PIFs) AtPIF3 and ZmPIF4 [35, 68]. We hypothesized that, BdSLR1 and BdSLRL1 could interact with BdGID1 (BRADI2G25600), BdPIF3 (BRADI2G11100) and BdPIF4 (BRADI1G13980) due to their high identities. Results suggested that both BdSLR1D and BdSLRL1 could interact with BdPIF3 and BdPIF4 (Fig. 8a, b). There were also weak interactions between BdGID1 with full, but not the truncated, BdSLR1 that could be strengthened by GA_3_ (Fig. 8c, d). This suggests that the interaction between BdSLR1 and BdGID1 also depends on the DELLA domain and the TVHYNP motif like OsSLR1 and OsGID1 [34, 69, 70]. Consistent with this hypothesis, BdSLRL1 did not interact with BdGID1 (Fig. 8b). As well, BdSLR1 and BdSLRL1 can form homo-dimers (Fig. 8a), but they could not interact with each other (Additional file 10: Figure S9). Bimolecular florescence complementation (BiFC) assay further verified these interactions (Fig. 8e). The protein interaction activity and transactivation activity of BdSLR1 and BdSLRL1 were similar to their homologs in Arabidopsis, rice and maize [34, 35, 68–70], indicating that orthologs with same motifs may have conserved functions among different species. Using this enables us to predict the functions of unknown genes in a variety of plant species.

## Discuss**i**on

GRAS transcription factors have been investigated widely among plants [71]. In this study, 48 *GRAS* genes were identified from *Brachypodium distachyon* genome. Among them, 7 (14.6%) and 14 (29.2%) genes were identified as tandem duplicated and segmental duplicated genes respectively. This corresponded with those in Arabidopsis (2 tandem duplicated genes/16 segmental duplicated genes/34 genes in total) [72], rice (10/8/45) [72], maize (11/22/86) [44] and *Prunus mume* (10/14/46) [45] and indicated that gene duplications play a role in the expansion of GRAS gene family in both monocots and dicots [40, 45, 53, 73]. These duplicated genes might undergo non-functionalization, neo-functionalization or sub-functionalization during evolution process that could generate alternative functions [74].

Genome-wide identification of *GRAS* genes has been reported in many plants including dicots such as Arabidopsis [26], tomato [40], and Chinese cabbage [53], monocots including rice [26, 41] and maize [44], and so on. According to phylogenic analyses, *GRAS* genes were divided into ten main subfamilies: SCR, SHR, DELLA, SCL3, PAT1, LlSCL (SCL9), SCL4/7, HAM, DLT and LAS [44, 75]. Based on the clade support values and the classification of homologs in rice and maize, *BdGRAS* genes were also divided into the same ten subfamilies.

The expression of *BdGRAS* genes in some subfamilies were similar to their homologs in other species. For example, in subfamily PAT1, most genes showed higher expression in leaves and roots that matched their orthologs in Arabidopsis [76], rice [26], castor bean [77] and sacred lotus [58]. Members of SCL3 were mainly expressed in inflorescence which is consistent with their homologs in Arabidopsis [76] and castor beans [77]. *LAS* gene was expressed in all four tissues, similar to its homologs in Arabidopsis [76]. *BdSCR* genes showed higher expressed in roots and leaves, consistent with those in rice [26], sacred lotus [58] and castor beans [77]. Conserved expression profiles of *GRAS* genes in different species indicate that homologous genes might have related functions in the development of these tissues. While the expression levels of *BdGRAS* genes in responses to different abiotic stresses and phytohormones were quite different even among members of the same subfamily. This is consistent with those in tomato [40], *Populus* [41] and *Prunus mume* [45], suggesting their different roles in abiotic stress responses and hormone-mediated signal pathways.

Similar to their homologs in Arabidopsis [26], rice [26], Chinese cabbage [53] and maize [44], *BdGRAS* genes in the same subfamilies showed conserved amino acid sequences and conserved motifs, which might imply conserved functions. Based on reports of functionally characterized genes, this might enable us to predict the functions of unknown proteins.

In most cases, *GRAS* members on the same branches showed similar functions (Additional file 1: Table S12). For example, *SCARECROW* genes in Arabidopsis, rice and maize function in the radial patterning of roots and shoots [3, 4, 78, 79]. Arabidopsis *LAS* and tomato *Lateral suppressor* are members of the LAS subfamily. Their mutations led to strong defects in axillary shoot meristem initiation [8, 10]. Similarly, mutations in the rice ortholog *MOC1*, resulted in few tillers, rachis branches and spikelets [9]. Arabidopsis, pepper, tomato and petunia *HAM* genes showed conserved function in the maintenance and organization of shoot apical meristem [7, 11–13]. In *Brachypodium distachyon*, the *GRAS* gene *BdSHR* was reported to play similar roles with its orthologs, *AtSHR* and *OsSHR*, in the regulation of meristem and root growth [59]. However, functions of other *BdGRAS* genes have not been reported. More effort needs to be made to characterize the functions of *BdGRAS* genes.

According to GO analyses and the KEGG pathway, two *DELLA* genes *BdSLR1* and *BdSLRL1* likely participate in the GA mediated signaling pathway. As reported previously, *DELLA* genes negatively regulate GA signals, including *GAI*, *RGA* and *RGL1/2/3* in Arabidopsis [1, 2], *SLR1* in rice [18], *SLN1* in barley [19], *DWARF-8* and *DWARF-9* [20, 80] in maize, and *Rht-B1/Rht-D1* in wheat [20]. Nevertheless, the functions of *DELLA*s in *Brachypodium distachyon* have not been verified. In this study, we showed the conserved functions of two *DELLA* genes *BdSLR1* and *BdSLRL1* in plant growth via GA signals.

Both transgenic plants over-expressing *BdSLR1* and *BdSLRL1* displayed dwarfism and late-flowering phenotypes which was consistent with their orthologs [1, 2, 20, 28, 34, 64, 65]. In addition, over-expression of *BdSLRL1* resulted in transgenic plants insensitive to exogenous GA_3_ like their orthologs in rice *OsSLRL1* [64] and *OsSLRL2* [65]. Sequence analyses showed that BdSLR1 contains a DELLA domain and a TVHYNP motif at the N-terminal, and a conserved GRAS domain at the C-terminal. BdSLRL1 has only a conserved GRAS domain at the C-terminal (Fig. 7). In rice, the suppressive function of SLR1 depends on the conserved GRAS domain; the N-terminal including the DELLA domain and the TVHYNP motif acts as the GA signal perception domain [28]. The repressive function of both BdDELLA proteins may also be due to the conserved GRAS domain while the difference between BdSLR1 and BdSLRL1 in GA signal perception may depend on the existence of the DELLA domain and the TVHYNP motif at the N-terminal of BdSLR1.

In Arabidopsis, RGA can physically interact with AtPIF3 and ZmPIF4 in the absence of GA and negatively controls PIFs-mediated hypocotyl elongation [35, 68]. After application of GA_3_, GIBBERELLIN INSENSITIVE DWARF1 (GID1), a GA receptor, binds to GA_3_ and then blocks the repression activity of DELLA proteins by directly interacting with the DELLA domain [36] or starting DELLA ubiquitylation by SCF^SLY1^ E3 ligase to initiate their degradation [66, 67, 69]. Thus, PIFs were released to promote plant growth. BdSLR1 and BdSLRL1 could also interact with BdPIF3 and BdPIF4 (Fig. 8). In addition, BdSLR1 could interact with BdGID1 and this interaction could be strengthened by GA_3_ (Fig. 8).

In *35S-BdSLR1* transgenic Arabidopsis, exogenous GA_3_ strengthened the interaction between GID1 and BdSLR1 (Fig. 8). This could restrain repressor activity and release PIFs from BdSLR1-PIF complexes. As a result, the phenotypes were recovered by application of exogenous GA_3_. As opposed to BdSLR1, BdSLRL1 could not interact with GID1, but could interact with BdPIFs (Fig. 8). When BdSLRL1 is excessively accumulated, the BdPIFs are restrained and affect growth. Since GID1 does not interact with BdSLRL1, the GA_3_-GID1 complex could not compete BdSLRL1 with PIFs. Thus, exogenous GA_3_ could not recover the phenotype. Further research should be able to verify these predictions and reveal vital mechanisms.

Our results showed that BdSLR1 and BdSLRL1 play a role in plant growth via negatively regulating the GA signal in a conserved manner similar to their orthologs [1, 2, 18–20, 80]. This supported the prediction that genes in the same branch may play conserved functions.

## Conclusions

We identified 48 *GRAS* genes in the *Brachypodium distachyon* genome and classified them into ten subfamilies using phylogenic analyses. Bioinformatics analyses and expression profiles indicate different GRAS proteins have different functions, while the members in same subfamily likely have similar functions. This was supported by the conserved functions of both *BdSLR1* and *BdSLRL1* genes in plant development via negatively regulating GA signals.

## Methods

### Genome-wide identification of *BdGRAS* genes

Sequences of genome DNA, CDS and proteins of *Brachypodium distachyon* (Bd21) (assembly v2.0) and *Triticum aestivum* (Chinese Spring) (assembly iwgsc_refseqv1.0) were obtained from http://www.gramene.org/. Protein sequences of the GRAS family in maize and sorghum were downloaded from the Plant Transcription Factor Database (PlantTFDB v4.0) [81]. Multiple alignments were performed using MEGA software (v6.0) (choosing ‘align by clustalW’ option with default parameters) [52].

The GRAS family Hidden Markov Model (HMM) profile (PF03514) was downloaded from the Pfam database (http://pfam.xfam.org/, v31.0). A second GRAS HMM profile was built by HMMER (v3.0) based on the multiple sequence alignment of conserved GRAS domains of AtGRAS proteins (downloaded from http://www.Arabidopsis.org/, release 10.0) and OsGRAS proteins (downloaded from http://rice.plantbiology.msu.edu/, release 7.0) [75]. Both GRAS HMM profiles were used as the query to perform the hmmsearch by HMMER (v3.0) against the annotated protein database of *Brachypodium distachyon* and wheat with a cut-off expected value (E-value) of 10^− 5^.

All hits identified by two HMM profiles were compared, and consensus sequences were retained. SMART sequence analysis [82] with a threshold of E-value less than 10^− 5^ was conducted among these candidate proteins to exclude those lacking the GRAS domain. BlastN in expressed sequence tags (EST) with a threshold of E-value less than 10^− 5^ and identity above 50% was also applied to support our identifications (http://www.ncbi.nlm.nih.gov).

### Analyses of protein properties, GO annotations, KEGG pathways, and phylogenetic relationship

Molecular weight (MW) and the theoretical isoelectric point (PI) values were calculated by ExPASy (https://web.expasy.org/protparam/). The GO (gene ontology) annotations were obtained from Monocots PLAZA v4.0 (https://bioinformatics.psb.ugent.be/plaza/) and Gramene v3.0 and then analyzed by BGIWEGO (v2.0) [83]. The Kyoto Encyclopedia of Genes and Genomes (KEGG) pathway of *BdGRAS* genes were analyzed online using protein sequences (https://www.genome.jp/kegg/pathway.html). An un-rooted neighbor joining (NJ) tree of GRAS proteins from rice, wheat, maize, sorghum and *Brachypodium distachyon* was constructed using MEGA (v6.0) [52] with 1000 bootstrap replications and annotated by Evolview (v2.0) [84].

### Synteny, *cis*-elements, gene structures and conserved motifs analyses of *BdGRAS* genes

Syntenic gene pairs among *Brachypodium distachyon*, and between rice, maize, sorghum, wheat and *Brachypodium distachyon* were identified using the Multiple Collinearity Scan toolkit (MCScanX) with default parameters [47]. The *Brachypodium* gene set was used as the chromosomal reference. Chromosomal distributions of *GRAS* genes were obtained from genome annotations and visualized using Circos (v 0.69) along with duplicated gene pairs.

The synonymous substitution (Ks) and non-synonymous substitution (Ka) rates were calculated by KaKs_calculator (v2.0) using the NG method [85]. Ks values were used to calculate the dates of duplication events (T) using the formula T = Ks/2λ × 10^− 6^ (millions of year, Mya) [51] assuming universal clock-like rate for *Brachypodium distachyon* was 6.1 × 10^− 9^ substitutions per synonymous site per year [86].

The 1.5 kb genomic DNA sequences in the 5′ flanking region of *BdGRAS* genes were downloaded from NCBI and then submitted to the PlantCARE for *cis-*elements analysis. The intron-exon organizations were analyzed through the Gene Structure Display Server v2.0 (http://gsds.cbi.pku.edu.cn/). MEME server v5.0.4 was applied to detect the conserved motifs with maximum number of 20 and optimum width of 5–200 amino acids. Gene structures and conserved motifs were visualized using Evolview (v2.0) [84].

### Stress and phytohormone treatments of *Brachypodium distachyon* and quantity RT-PCR

Two-week-old *Bd21* seedlings were put in a Murashige and Skoog (MS) liquid medium containing 200 mM NaCl, 20% PEG6000, 10 mM H_2_O_2_, 1 mM SA, 100 μM MeJA, 100 μM ABA, 20 μM 6-BA and 3 μM GA for 2 h, respectively, to mimic salt, drought, oxidative stresses and phytohormone stimulation. Seedlings were placed in a 45 °C or 4 °C climate chamber for 2 h to imitate heat or cold stresses. Seedlings with no treatment served as control. The leaves and roots of Bd21 were collected separately after treatment. Roots, stems, leaves and inflorescences were acquired from plants during the heading period. All materials were flash frozen by liquid nitrogen and stored at − 80 °C until analysis.

Total RNA was extracted using the TRIZOL reagent (TAKARA) and treated with RNase-free DNase I (TAKARA) according to the manufacturer’s instructions. A reverse transcription reaction using total RNA (above) was carried out as described previously with a Transcriptor First Strand cDNA Synthesis Kit (Roche) [87]. qRT-PCR reaction were performed by a QuantStudio 7 Flex Real-Time PCR System (ThermoFisher Scientific) in triplicate with 15 μl reaction mixture consisting of 7.5 μl SYBR® Premix Ex Taq (TAKARA), 0.5 μl cDNA (5.0 ng/μl), 0.3 μl ROX reference Dye (50×),1.5 μl (10 pmol/μl) forward primer, 1.5 μl (10 pmol/μl) reverse primer, and 3.7 μl ddH_2_O. The qRT-PCR event sequence was: preheat at 50 °C for 2 min, predenaturation at 95 °C for 10 min, 40 cycles of PCR reactions at 95 °C for 15 s and 60 °C for 1 min with fluorescence being measured at the end of each cycle, melt curve at 95 °C for 15 s, 60 °C for 1 min, and 95 °C for 15 s with fluorescence being measured during the heating period from 60 °C to 95 °C. Relative expression levels of target genes (primers used in this study were designed by Primer Premier v5.0 [88] and listed in Additional file 1: Table S8) were calculated by the 2^(−ΔΔCt)^ analysis method [89]. Ct means were normalized with the expression of *GAPDH* in *Brachypodium distachyon* (*BRADI3G14120*) [90] and Arabidopsis (*AT1G13440*) [91].

### 5′-rapid amplification of cDNA ends (5′-RACE)

Total RNA extracted from inflorescences of *Brachypodium distachyon* was used to produce the first strand cDNA with Rapid Amplification of cDNA Ends kit (TAKARA) using the manufacturer’s instructions. Primers for 5′-RACE (Rapid Amplication of cDNA Ends) of *BRADI2G45117* were designed using Primer Premier v5.0 [88] based on its CDS sequence downloaded from Gramene. 5′-RACE was conducted using primers RACEPR1 (used in the first PCR) and RACEPR2 (second PCR) (Additional file 1: Table S8) with the following PCR conditions: 95 °C for 5 min, 40 cycles (95 °C for 30s, 58 °C for 30s, 72 °C for 1 min), 72 °C for 10 min. Then the full length sequence of *BRADI2G45117* containing the 5′-UTR and CDS was amplified using rTaq (Sangon) according to the manufacturer’s protocols with cDNA from inflorescence of *Brachypodium distachyon* as the template and BdSLRL1FLPF and BdSLRL1FLPR as primers. The PCR process was as follows: 98 °C for 5 min, 40 cycles (98 °C for 45 s, 58 °C for 45 s, 72 °C for 2 min), 72 °C for 10 min.

### Plasmid construction, yeast two-hybrid assay, BiFC and plant transformation

The amplified fragments with additional Nde I and EcoR I sites through corresponding primers (Additional file 1: Table S8) were cloned separately into the DNA binding vector pGBKT7 and activation domain vector pGADT7. Recombined vectors were transformed into the yeast strain Y2H using the LiAc transformation method [92] and coated on synthetic dextrose (SD) -Trp or SD-Trp-Leu for growth tests. Yeast clones were plated on SD-His-Trp-Ade and SD-His-Trp-Leu-Ade medium for 3 days at 30 °C to assay for self-activation and protein interaction.

The coding sequence were amplified using primers listed in Additional file 1: Table S8 and then introduced into vectors p1302-eYFP-N and p1302-eYFP-C using recombination reactions. Recombined plasmids were transformed into the *Agrobacterium tumefaciens* strain GV3101 and then co-infiltrated with *Agrobacterium* carrying the p19 silencing plasmid into leaves of 1-month-old *Nicotiana benthamiana* plants. Two days after infiltration, eYFP signals were observed with a fluorescence microscopy (Olympus IX83-FV1200).

The coding sequence were cloned with primers in Additional file 1: Table S8 and ligated into the over-expression vector pCAMBIA1300 using recombination reactions. Recombined plasmids were introduced into GV3101 and then transformed into Arabidopsis Col-0 via the flowerer-dipping method [93].

### Arabidopsis materials and treatments

Plants were grown in long-day conditions of 22 °C, 16 h light/20 °C, 8 h dark cycles. Transgenic lines were selected using 1/2MS medium containing 40 mg/L hygromycin B. For the GA treatment, surface-sterilized seeds were vertically cultivated on 1/2MS medium with or without 10 μM GA_3_ for 7 days. Photographs were then taken and seedlings were collected for gene expression. Hypocotyls of at least 30 seedlings were measured via ImageJ and data was analyzed with SPSS (IBM SPSS Statistics 20).

## Additional files

Additional file 1 **: Table S1.** The chromosome location and physicochemical characteristics of *BdGRAS* genes. **Table S2.** The Ka and Ks values and estimated divergence time for tandemly duplicated *BdGRAS* genes. **Table S3.** The Ka and Ks values and estimated divergence time for segmentally duplicated *BdGRAS* genes. **Table S4.** The chromosome location, Ka and Ks values, and estimated divergence time for orthologous *GRAS* genes between *Brachypodium* and rice. **Table S5.** The chromosome location, Ka and Ks values, and estimated divergence time for orthologous *GRAS* genes between *Brachypodium* and sorghum. **Table S6.** The chromosome location, Ka and Ks values, and estimated divergence time for orthologous *GRAS* genes between *Brachypodium* and maize. **Table S7.** The chromosome location, Ka and Ks values, and estimated divergence time for orthologous *GRAS* genes between *Brachypodium* and wheat. **Table S8.** The primers used in this study. **Table S9.** GO annotations of BdGRAS proteins. **Table S10.** GO descriptions for *BdGRAS* proteins. **Table S11.** Numbers of known *cis-*elements in the promoter regions of *BdGRAS* genes. **Table S12.** Functions of *GRAS* genes in other species. (XLSX 163 K) (XLSX 162 kb)

Additional file 2: **Figure S1.** Alignment of BdGRAS proteins to show conserved domains and amino acids. (JPG 9.5 M) (JPG 9755 kb)

Additional file 3: **Figure S2.** Amino acid sequence of conserved motifs identified by MEME. The font size represents the frequency of each amino acid. (JPG 3.8 M) (JPG 3895 kb)

Additional file 4: **Figure S3.** Agarose gel electrophoresis results of *BRADI2G45117* 5′-RACE (second PCR). (TIF 1.03 M) (TIF 1055 kb)

Additional file 5: **Figure S4.** DNA sequencing results of *BRADI2G45117* 5′-UTR. (TIF 1.9 M) (TIF 1970 kb)

Additional file 6: **Figure S5.** Agarose gel electrophoresis results of *BRADI2G45117* full length (including 5′-UTR and CDS) PCR. (JPG 406 K) (JPG 406 kb)

Additional file 7: **Figure S6.** DNA sequencing results of *BRADI2G45117* full length (including 5′-UTR and CDS) PCR using forward primer BdSLRL1FLPF. (JPG 2.3 M) (JPG 2311 kb)

Additional file 8: **Figure S7.** DNA sequencing results of *BRADI2G45117* full length (including 5′-UTR and CDS) PCR using reverse primer BdSLRL1FLPR. (JPG 2.4 M) (JPG 2456 kb)

Additional file 9: **Figure S8.** Sequence analyses of BRADI2G45117 (including 5′-UTR and CD). 5′-UTR are in grey. Start codon and stop codon are in red and blue, respectively. Full length PCR primers, 5′-RACE GSP outer primer and inner primer are underlined with dashed lines, full lines and wavy lines, respectively. (PDF 17 K) (PDF 16 kb)

Additional file 10: **Figure S9.** Yeast two hybrid and transactivation activities assays of BdSLR1 and BdSLRL1. (TIF 14.7 M) (TIF 15054 kb)

Additional file 11: **Text 1.** Synteny gene pairs between rice and *Brachypodium distachyon*. (TXT 904 K) (TXT 903 kb)

Additional file 12: **Text 2.** Synteny gene pairs between rice and *Brachypodium distachyon*. (TXT 1.77 M) (TXT 1818 kb)

Additional file 13: **Text 3.** Synteny gene pairs between rice and *Brachypodium distachyon*. (TXT 1.28 M) (TXT 1320 kb)

Additional file 14: **Text 4.** Synteny gene pairs between rice and *Brachypodium distachyon*. (TXT 2.59 M) (TXT 2661 kb)

Additional file 15: **Text 5.** Protein sequences of GRAS in rice, maize, sorghum, wheat and *Brachypodium distachyon*. (TXT 270 K) (TXT 270 kb)

## Data Availability

The GRAS gene family datasets analyzed in this article are included within this article and supplementary files (the Additional files 11-15), and the rests are available from the corresponding author on reasonable request.
