# Supplementary figures and images for: Genome-wide identification of GRAS genes in Brachypodium distachyon and functional characterization of BdSLR1 and BdSLRL1
Source: BMC Genomics. 2019 Aug 6;20:635. doi: 10.1186/s12864-019-5985-6 (PMC6683515; doi:10.1186/s12864-019-5985-6)

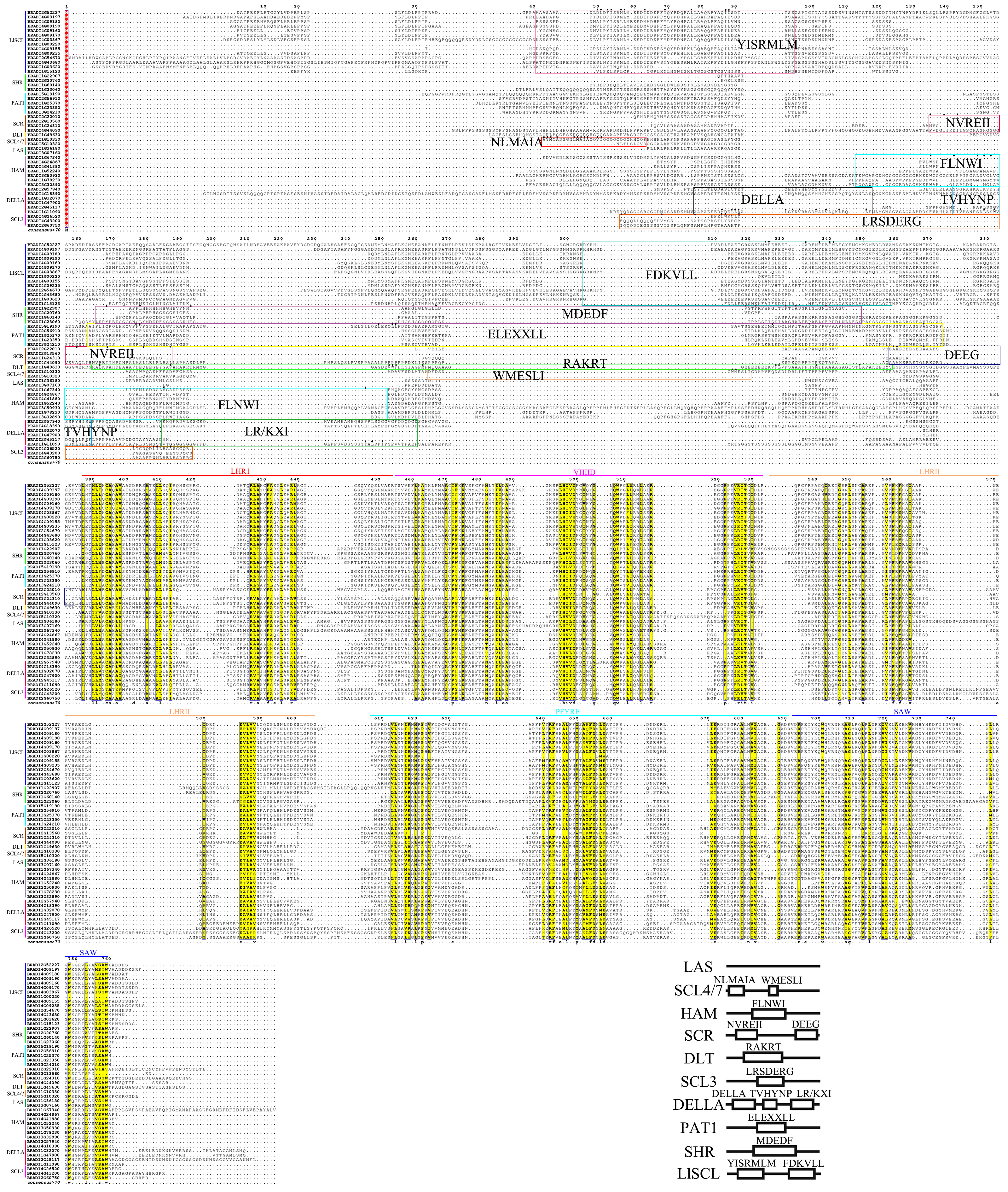

Supplement: Supplementary file 2 — Figure S1. Alignment of BdGRAS proteins to show conserved domains and amino acids. (JPG 9.5 M) (JPG 9755 kb) [file 12864_2019_5985_MOESM2_ESM.jpg]

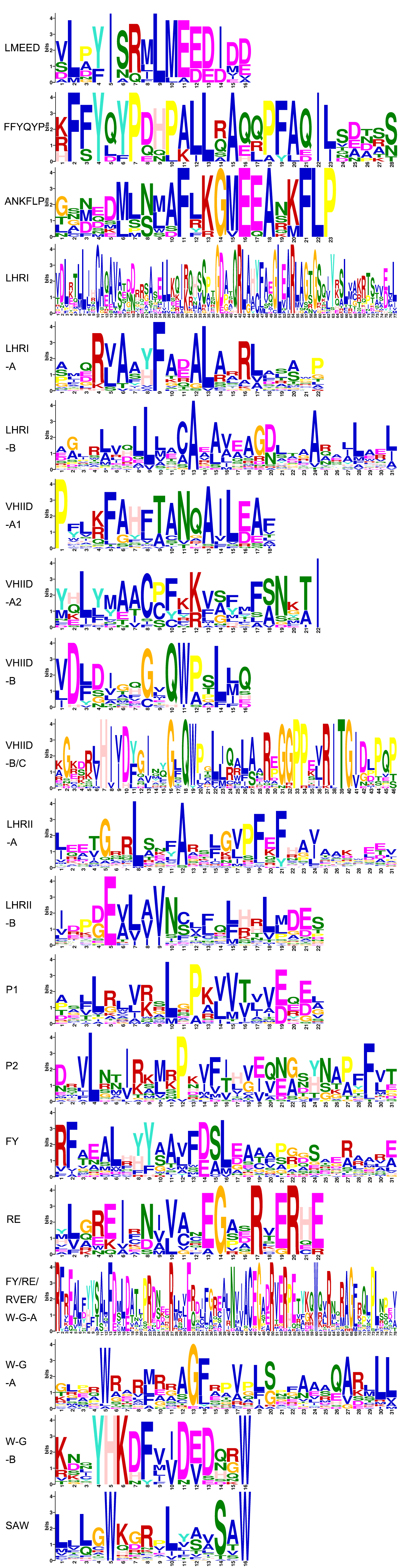

Supplement: Supplementary file 3 — Figure S2. Amino acid sequence of conserved motifs identified by MEME. The font size represents the frequency of each amino acid. (JPG 3.8 M) (JPG 3895 kb) [file 12864_2019_5985_MOESM3_ESM.jpg]

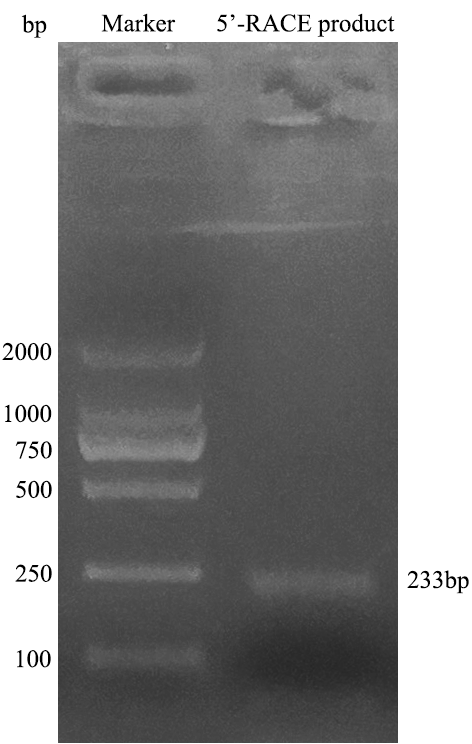

Supplement: Supplementary file 4 — Figure S3. Agarose gel electrophoresis results of BRADI2G45117 5′-RACE (second PCR). (TIF 1.03 M) (TIF 1055 kb) [file 12864_2019_5985_MOESM4_ESM.tif]

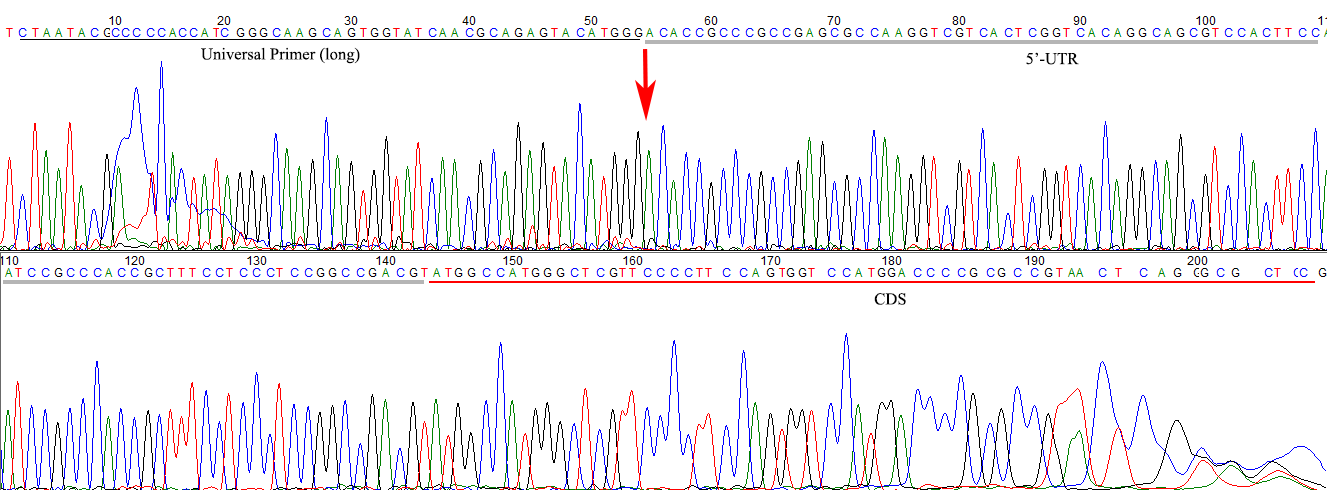

Supplement: Supplementary file 5 — Figure S4. DNA sequencing results of BRADI2G45117 5′-UTR. (TIF 1.9 M) (TIF 1970 kb) [file 12864_2019_5985_MOESM5_ESM.tif]

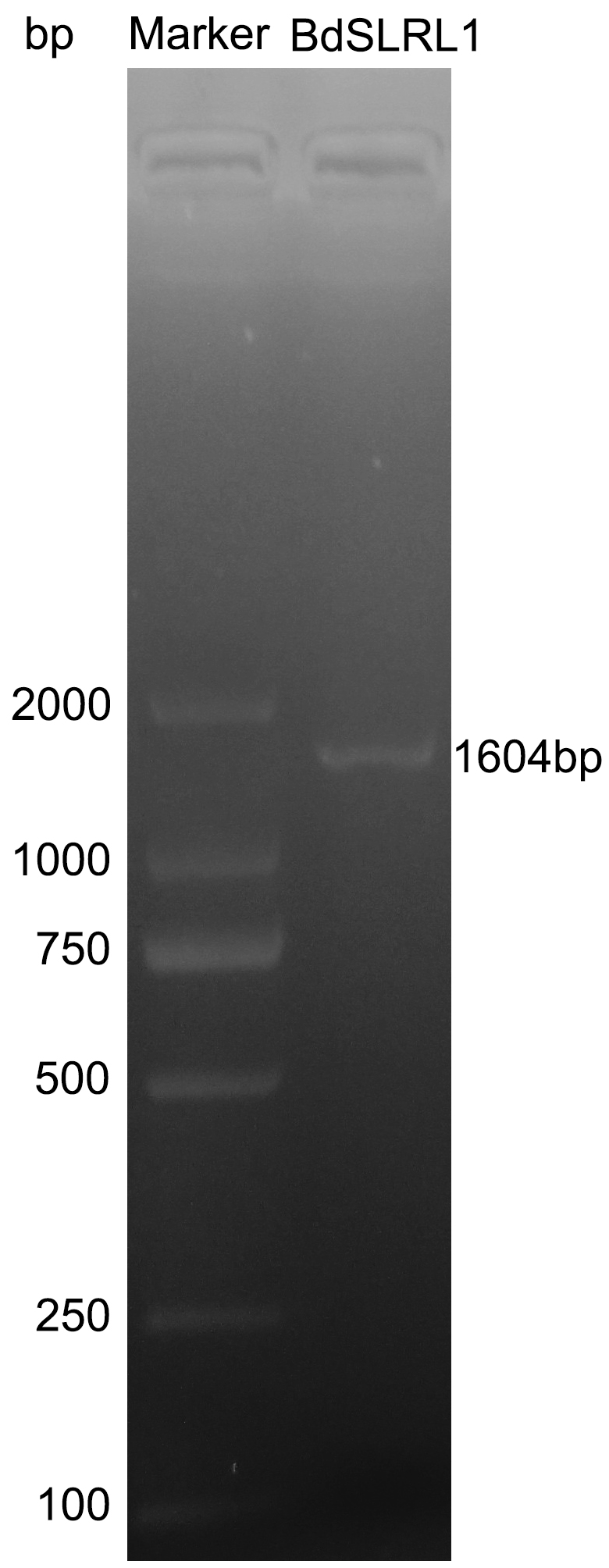

Supplement: Supplementary file 6 — Figure S5. Agarose gel electrophoresis results of BRADI2G45117 full length (including 5′-UTR and CDS) PCR. (JPG 406 K) (JPG 406 kb) [file 12864_2019_5985_MOESM6_ESM.jpg]

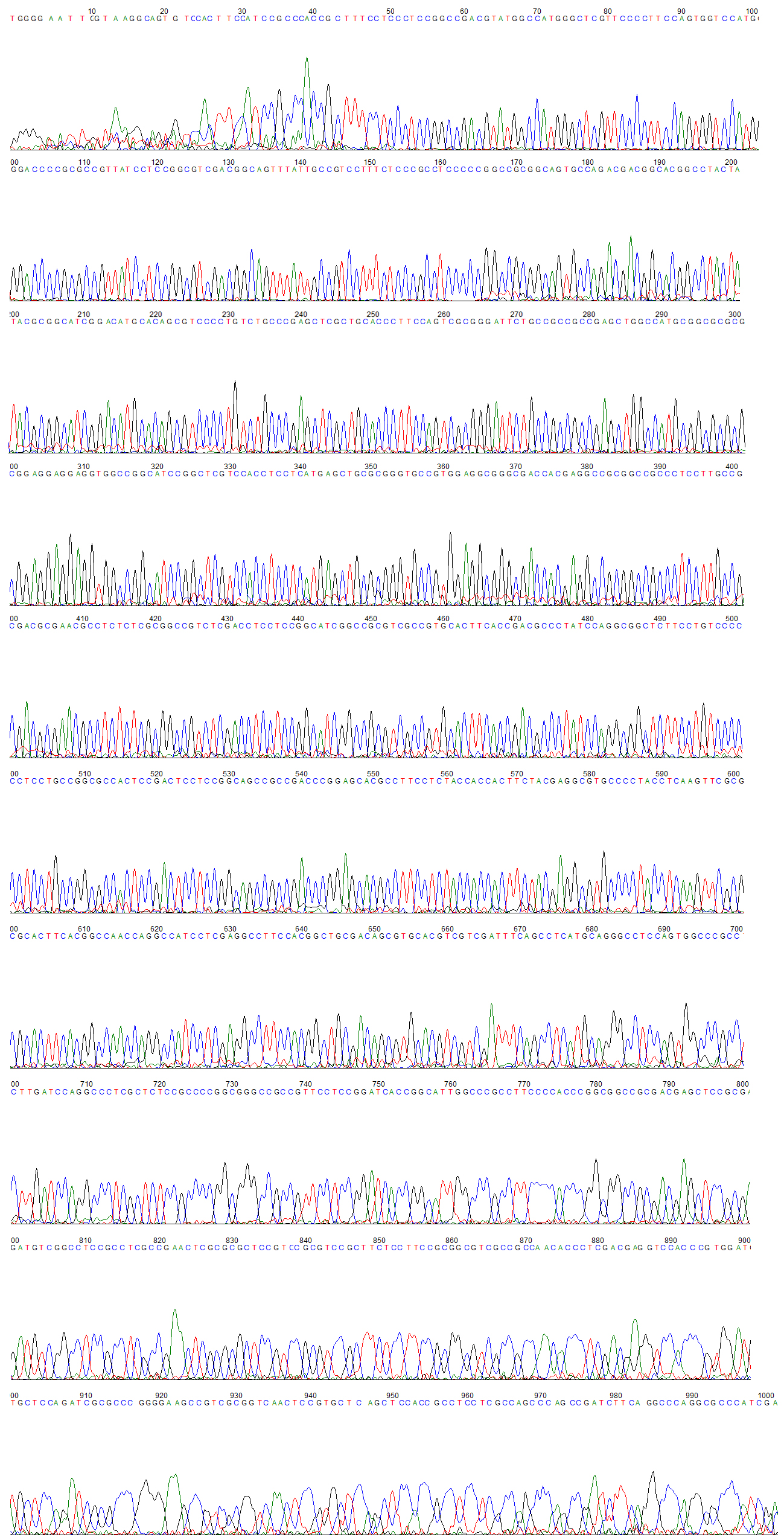

Supplement: Supplementary file 7 — Figure S6. DNA sequencing results of BRADI2G45117 full length (including 5′-UTR and CDS) PCR using forward primer BdSLRL1FLPF. (JPG 2.3 M) (JPG 2311 kb) [file 12864_2019_5985_MOESM7_ESM.jpg]

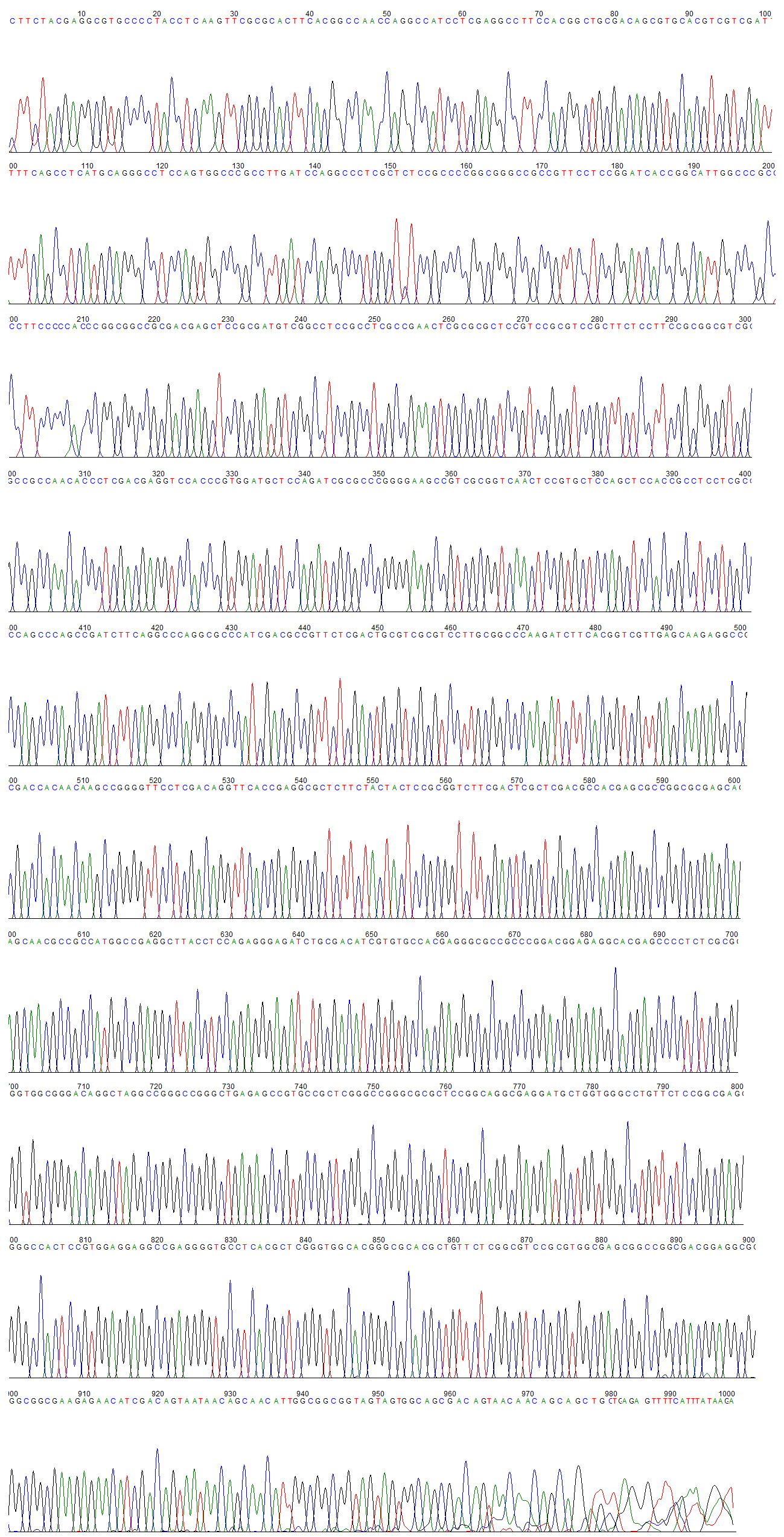

Supplement: Supplementary file 8 — Figure S7. DNA sequencing results of BRADI2G45117 full length (including 5′-UTR and CDS) PCR using reverse primer BdSLRL1FLPR. (JPG 2.4 M) (JPG 2456 kb) [file 12864_2019_5985_MOESM8_ESM.jpg]

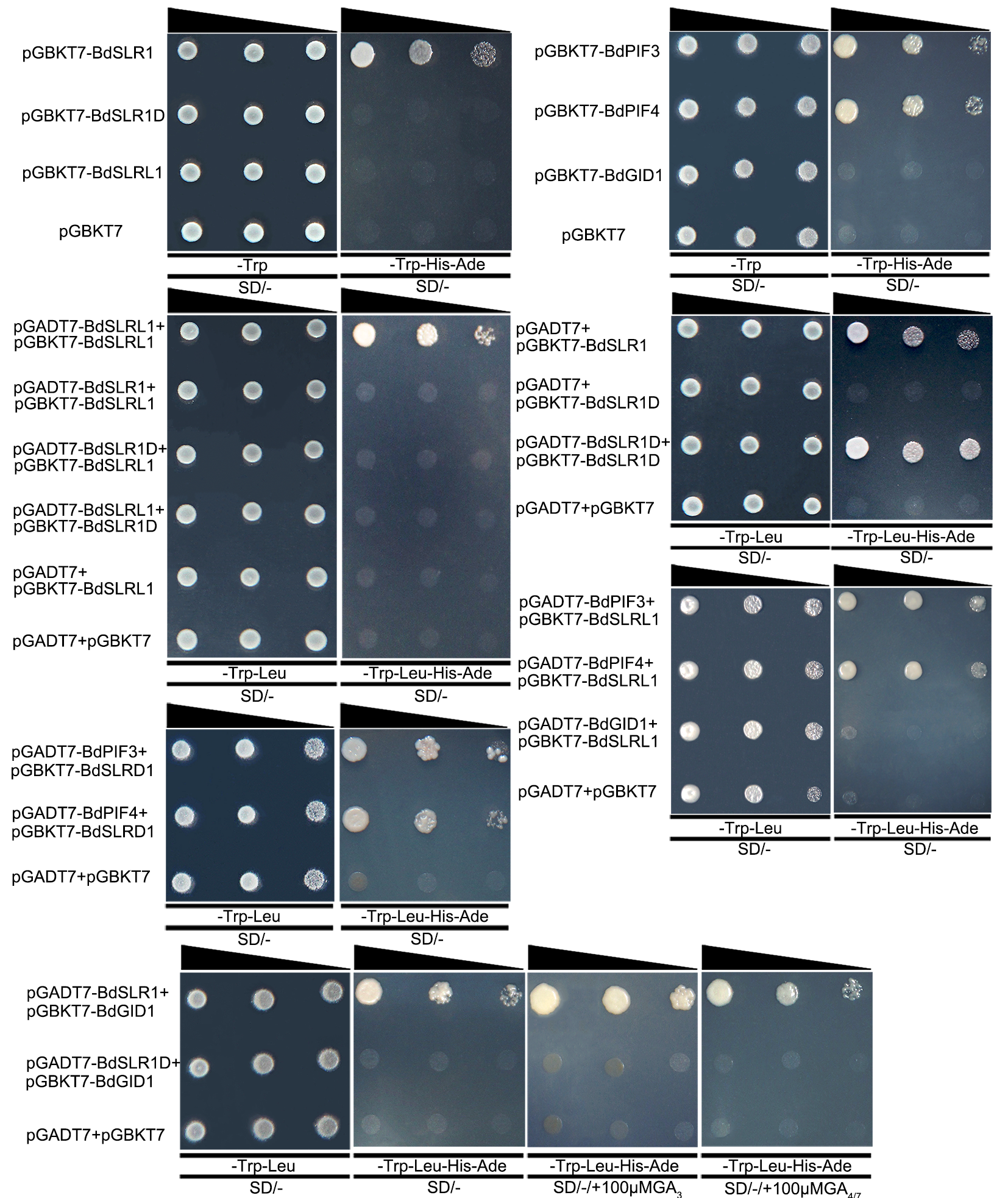

Supplement: Supplementary file 10 — Figure S9. Yeast two hybrid and transactivation activities assays of BdSLR1 and BdSLRL1. (TIF 14.7 M) (TIF 15054 kb) [file 12864_2019_5985_MOESM10_ESM.tif]
