# Supplementary material for: Genome-wide identification of GRAS genes in Brachypodium distachyon and functional characterization of BdSLR1 and BdSLRL1
Source: BMC Genomics. 2019 Aug 6;20:635. doi: 10.1186/s12864-019-5985-6 (PMC6683515; doi:10.1186/s12864-019-5985-6)
Supplement: Supplementary file 9 — Figure S8. Sequence analyses of BRADI2G45117 (including 5′-UTR and CD). 5′-UTR are in grey. Start codon and stop codon are in red and blue, respectively. Full length PCR primers, 5′-RACE GSP outer primer and inner primer are underlined with dashed lines, full lines and wavy lines, respectively. (PDF 17 K) (PDF 16 kb) [file 12864_2019_5985_MOESM9_ESM.pdf]

ACACCGCCCGCCGAGCGCCAAGGTCGTCACTCGGTCACAGGCAGCGTCCACTTCCATCCGCCCACCGCT
TTCCTCCCTCCGGCCGACGTATGGCCATGGGCTCGTTCCCCTTCCAGTGGTCCATGGACCCCGCGCCGT
TATCCTCCGGCGTCGACGGCAGTTTATTGCCGTCCTTTCTCCCGCCTCCCCCGGCCGCGGCAGTGCCAG
ACGACGGCACGGCCTACTACGCGGCATCGGACATGCACAGCGTCCCCTGTCTGCCCGAGCTCGCTGCAC
CCTTCCAGTCGCGGGATTCTGCCGCCGCCGAGCTGGCCATGCGGCGCGGAGGAGGAGGTGGCCGGCA
TCCGGCTCGTCCACCTCCTCATGAGCTGCGCGGGTGCCGTGGAGGCGGGCGACCACGAGGCCGCGGCCG
CCCTCCTTGCCGACGCGAACGCCTCTCTCGCGGCCGTCTCGACCTCCTCCGGCATCGGCCGCGTCGCCG
TGCACTTCACCGACGCCCTATCCAGGCGGCTCTTCCTGTCCCCTCCTGCCGGCGCCACTCCGACTCCTC
CGGCAGCCGCCGACCCGGAGCACGCCTTCCTCTACCACCACTTCTACGAGGCGTGCCCCTACCTCAAGT
TCGCGCACTTCACGGCCAACCAGGCCATCCTCGAGGCCTTCCACGGCTGCGACAGCGTGCACGTCGTCG
ATTTCAGCCTCATGCAGGGCCTCCAGTGGCCCGCCTTGATCCAGGCCCTCGCTCTCCGCCCCGGCGGGC
CGCCGTTCCTCCGGATCACCGGCATTGGCCCGCCTTCCCCACCCGGCGGCCGCGACGAGCTCCGCGATG
TCGGCCTCCGCCTCGCCGAACTCGCGCGCTCCGTCCGCGTCCGCTTCTCCTTCCGCGGCGTCGCCGCCA
ACACCCTCGACGAGGTCCACCCGTGGATGCTCCAGATCGCGCCCGGGGAAGCCGTCGCGGTCAACTCCG
TGCTCCAGCTCCACCGCCTCCTCGCCAGCCCAGCCGATCTTCAGGCCCAGGCGCCCATCGACGCCGTTC
TCGACTGCGTCGCGTCCTTGCGGCCCAAGATCTTCACGGTCGTTGAGCAAGAGGCCGACCACAACAAGC
CGGGGTTCCTCGACAGGTTCACCGAGGCGCTCTTCTACTACTCCGCGGTCTTCGACTCGCTCGACGCCA
CGAGCGCCGGCGCGAGCAGCAACGCCGCCATGGCCGAGGCTTACCTCCAGAGGGAGATCTGCGACATCG
TGTGCCACGAGGGCGCCGCCCGGACGGAGAGGCACGAGCCCCTCTCGCGGTGGCGGGACAGGCTAGGCC
GGGCCGGGCTGAGAGCCGTGCCGCTCGGGCCGGGCGCGCTCCGGCAGGCGAGGATGCTGGTGGGCCTGT
TCTCCGGCGAGGGCCACTCCGTGGAGGAGGCCGAGGGGTGCCTCACGCTCGGGTGGCACGGGCGCACGC
TGTTCTCGGCGTCCGCGTGGCGAGCGGCCGGCGACGGAGGCGGCGGCGAAGAGAACATCGACAGTAATA
ACAGCAACATTGGCGGCGGTAGTAGTGGCAGCGACAGTAACAACAGCAGCTGCGGGGTCGTTGGTGCTG
CCAACATGTTTTTGTAA
